# Hyperglycemia increases SCO-spondin and Wnt5a secretion into the cerebrospinal fluid to regulate ependymal cell beating and glucose sensing

**DOI:** 10.1371/journal.pbio.3002308

**Published:** 2023-09-21

**Authors:** Francisco Nualart, Manuel Cifuentes, Eder Ramírez, Fernando Martínez, María José Barahona, Luciano Ferrada, Natalia Saldivia, Ernesto R. Bongarzone, Bernard Thorens, Katterine Salazar

**Affiliations:** 1 Laboratory of Neurobiology and Stem Cells, NeuroCellT, Department of Cellular Biology, Faculty of Biological Sciences, University of Concepcion, Concepcion, Chile; 2 Center for Advanced Microscopy CMA BIO BIO, University of Concepcion, Concepcion, Chile; 3 Department of Cell Biology, Genetics and Physiology, University of Malaga, Málaga Biomedical Research Institute and Nanomedicine Platform (IBIMA-BIONAND Platform), Malaga, Spain; 4 Department of Anatomy and Cell Biology, College of Medicine, University of Illinois at Chicago, Chicago, Illinois, United States of America; 5 Center for Integrative Genomics, University of Lausanne, Lausanne, Switzerland; UCSD, UNITED STATES

## Abstract

Hyperglycemia increases glucose concentrations in the cerebrospinal fluid (CSF), activating glucose-sensing mechanisms and feeding behavior in the hypothalamus. Here, we discuss how hyperglycemia temporarily modifies ependymal cell ciliary beating to increase hypothalamic glucose sensing. A high level of glucose in the rat CSF stimulates glucose transporter 2 (GLUT2)-positive subcommissural organ (SCO) cells to release SCO-spondin into the dorsal third ventricle. Genetic inactivation of mice GLUT2 decreases hyperglycemia-induced SCO-spondin secretion. In addition, SCO cells secrete Wnt5a-positive vesicles; thus, Wnt5a and SCO-spondin are found at the apex of dorsal ependymal cilia to regulate ciliary beating. Frizzled-2 and ROR2 receptors, as well as specific proteoglycans, such as glypican/testican (essential for the interaction of Wnt5a with its receptors) and Cx43 coupling, were also analyzed in ependymal cells. Finally, we propose that the SCO-spondin/Wnt5a/Frizzled-2/Cx43 axis in ependymal cells regulates ciliary beating, a cyclic and adaptive signaling mechanism to control glucose sensing.

## Abstract 2

Hyperglycemia increases glucose concentrations in the cerebrospinal fluid (CSF), activating glucose sensing and feeding behavior in the hypothalamus. This study shows that raised CSF glucose levels induce release of SCO-spondin and Wnt5a, controlling ciliary beating in the ependymal cells.

## Introduction

Under hyperglycemic conditions, glucose concentration increases proportionally in the peripheral blood and hypothalamic cerebrospinal fluid (CSF) [1]; thus, hypothalamic neurons and glial cells induce metabolic signaling to achieve satiety [2]. Glucose-sensing activity occurs in the ventral third ventricle in a specialized hypothalamic region where glial cells (tanycytes) that contact the CSF do not exhibit ciliary beating [3,4]. Local movement of CSF depends on ependymal cell ciliary beating in other ventricular regions (medial and dorsal). Ciliary beating and CSF flow vary inside the third ventricle and appear to be regulated by circadian clock–dependent activity [3], ethanol concentration [5,6], and energy balance (the ATP/ADP ratio and Ca^2+^ concentration); a decrease in the glucose concentration (from 25 mM to 0.1 mM) induces a reversible increase in ciliary beating frequency [7]. Thus, increasing intracellular glucose concentrations and, consequently, ATP content should decrease fluid movement [7]. Furthermore, it has been proposed that in mice, melanin-concentrating hormone (MCH) can regulate ciliary beating in the ventral region of the third ventricle, but not in the dorsal ventricular area or at the entrance to the cerebral aqueduct (CA) [8]. Additionally, 1 report identified 3 distinct types of ependymal cells uniquely and specifically positioned within the third ventricle and classified based on their cilia beating frequency as type I (>60 Hz), type II (30 to 60 Hz), or type III (<30 Hz) [5]. In this study, alcohol had a profound effect on the beating frequency of ependymal cilia, resulting in a substantial decrease in fluid movement and volume replacement [5].

SCO-spondin is a glycoprotein with multiple thrombospondin type 1 repeats (TSRs) that is secreted by cells in the subcommissural organ (SCO), an epithelial structure strategically located on the roof of the third ventricle [9–11], and mainly forms Reissner’s fibers (RFs) [12]. Intracellularly, SCO-spondin is synthesized in elongated endoplasmic reticulum (ER) cisternae in SCO cells. Proteins are glycosylated and stored in secretory granules that accumulate in the apical zone of the cells, where SCO-spondin is released into the CSF. Extracellularly, secreted SCO-spondin forms a thin film of secretion deposited on the cilia and blebs of the SCO cells. Subsequently, this protein forms fibrillary aggregates or can also remain soluble in the CSF [13].

It has been reported that fibrillary-aggregated SCO-spondin (SCO-spondin that forms RFs) plays a critical role in the maintenance of a straight body axis and spine morphogenesis in zebrafish [10]. Additionally, SCO-spondin (the soluble and/or aggregated form) has been implicated in CSF homeostasis [14,15], neurogenesis [16–19], embryonic morphogenesis [20], and hydrocephalus [21] during prenatal or early postnatal brain development; however, the function of SCO-spondin (the soluble and/or aggregated form) in the adult mammalian brain is unknown, as the SCO is an enigmatic structure inside the brain [12,22]. Furthermore, the mechanism by which SCO-spondin aggregation or solubilization is modulated by physiological or pathophysiological condition(s) is unknown. In vitro, it has been suggested that ATP increases SCO-spondin secretion and that 5-hydroxytryptamine (5-HT) inhibits SCO-spondin activity [23]. Additionally, in bovine SCO cells, ATP increases the [Ca^2+^]i in approximately 85% of cells [24]. These effects are dose dependent and involve NK3 and P2Y2 receptors linked to G protein and phospholipase C activation [24].

Here, we report that SCO cells express glucose transporter 2 (GLUT2) with apical polarization. Since GLUT2 is a low-affinity transporter that is preferentially expressed in tissues with glucose-sensing activity, we increased the glucose concentration in CSF [1]. We demonstrated that SCO-spondin is secreted into CSF in response to hyperglycemic conditions and interacts with multiciliated ependymal cells. Thus, ependymal cells temporarily decrease ciliary beating and CSF flow. Additionally, we observed that SCO cells secrete multivesicular bodies (MVBs; CD63+ vesicles) and wingless-type MMTV integration site family member 5A (Wnt5a), which binds to ependymal cells that interact with Frizzled 2/receptor tyrosine kinase-like orphan receptor-2 (ROR2). Finally, changes in connexin distribution were also observed. We suggest the existence a hyperglycemic response system in the human brain that involves activation of a signaling pathway that includes Spondin-like proteins, Wnt5a, Frizzled-2, ROR2, and connexin-43 (Cx43) in ependymal cells.

## Results

### SCO cells express GLUT2, secrete SCO-spondin into the CSF, and release MVB-like secretory vesicles (SVs)

**Fig 1:**
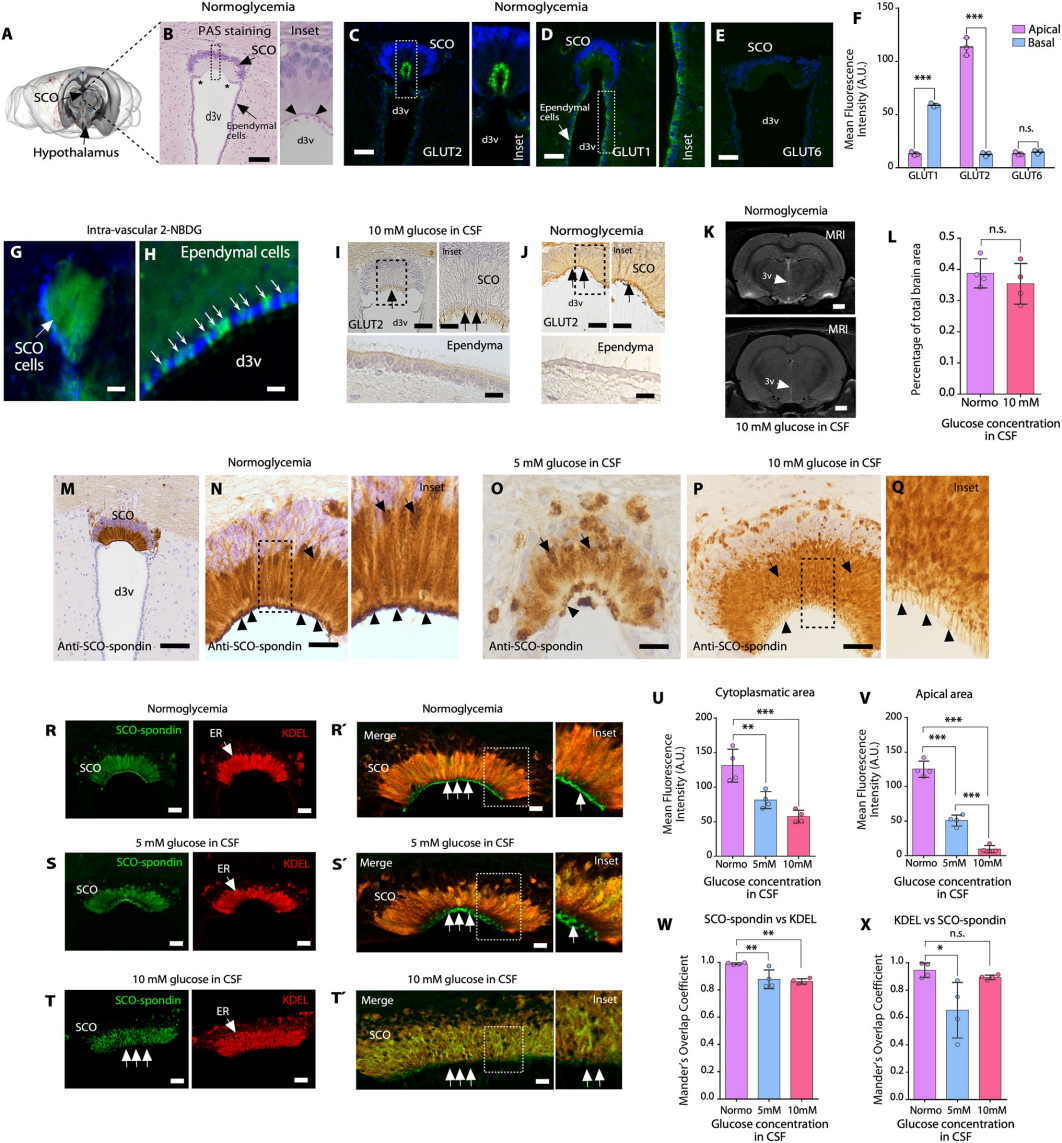
GLUT2 expression in SCO cells and SCO-spondin secretions into CSF under hyperglycemic conditions. (**A**) Representative image of the rat brain showing the location of the SCO. *Image credit*: *Allen Institute*. (**B**) Frontal brain sections containing SCO cells after PAS staining in the normoglycemic condition. Apical secretory granules are depicted (arrowheads in inset). Scale bar: 100 μm. (**C to E**) Immunohistochemical staining of GLUT2, GLUT1, and GLUT6 in frontal brain sections under normoglycemic conditions. Scale bar: 40 μm. (**F**) Quantitative analysis of GLUT1, GLUT2, and GLUT6 immunoreactivity in apical and basal areas of the SCO cells. The graph shows data from 3 biologically independent samples. The error bars represent the SD; ***P < 0.001, n.s. = not significant (two-tailed Student *t* test). (**G** and **H)** Intravascular 2-NBDG injection and SCO or ependymal cell analysis (*N* = 3). Scale bar: G, 100 μm; H, 20 μm. (**I, J**) Immunohistochemical staining of GLUT2 in frontal sections under hyperglycemic (30 min after intraperitoneal glucose injection) or normoglycemic conditions; the SCO and ependymal cells are depicted. GLUT2 reactivity was detected in the apical region of SCO cells (black arrows). *N* = 3. Scale bar: I and J, 25 μm. (**K**, **L**) Representative T1-weighted coronal MRI scans showing that the lateral and third ventricles were not enlarged in normoglycemic rats compared with hyperglycemic rats (CSF glucose concentration of 10 mM). The white arrows point to the d3v. Scale bar, 1 mm. (**L**) Percentage analysis of the area of the third ventricle in relation to the total area of the brain. The graph shows data from 4 biologically independent samples. The error bars represent the SD; n.s. = not significant (two-tailed Student *t* test). (**M**-**Q**) Immunohistochemical staining of SCO-spondin under normoglycemic conditions (M, N and inset) and hyperglycemic conditions, i.e., when the CSF glucose concentration was 5 mM (**O**) or 10 mM (**P, Q**) (30 min after intraperitoneal glucose injection). The apical region of SCO cells is indicated by the arrowheads. *N* = 3. Scale bar: M, 200 μm; N to P, 50 μm. (**R** and **Rˊ**) Immunofluorescence and confocal analysis of SCO-spondin and KDEL (ER marker) expression, in SCO cells under normoglycemic conditions. *N* = 3. Scale bar: R, 40 μm; Rˊ, 10 μm. (**S, T**) Immunofluorescence and confocal analysis of SCO-spondin and KDEL expression, in SCO cells under 5 mM glucose in CSF (S and Sˊ) or 10 mM glucose in CSF (T and Tˊ). *N* = 3. Scale bar: S and T, 40 μm; Sˊ and Tˊ, 10 μm. (**U**, **V**) Quantitative analysis of SCO-spondin immunoreactivity (apical and cytoplasmic areas of the cells) under different glycemic conditions. The graph shows data from 4 biologically independent samples. The error bars represent the SD; **P < 0.01, ***P < 0.001 (one-way ANOVA with Tukey’s posttest). (**W, X**) Quantification of Mander’s overlap coefficient for SCO-spondin vs. KDEL or KDEL vs. SCO-spondin in SCO cells under different glycemic conditions. The graph shows data from 4 biologically independent samples. The error bars represent the SD; *P < 0.05, **P < 0.01, n.s. = not significant (one-way ANOVA with Tukey’s posttest). Data used to generate graphs can be found in S1 Data. CSF, cerebrospinal fluid; d3v, dorsal third ventricle; ER, endoplasmic reticulum; GLUT, glucose transporter; PAS, Periodic acid–Schiff; SCO, subcommissural organ; 2-NBDG, D-glucose analog 2-[N-(7-nitrobenz-2-oxa-1,3-diazol-4-yl) amino]-2-deoxy-D-glucose.

SCO elongated cells were found lining the roof of the rat third cerebral ventricle (Fig 1A and 1B). These cells polarize their nuclei (purple zone) to the basal region of the epithelial cells and project their cytoplasm (abundant in ER and Golgi apparatus) to an apical region, which is in contact with the CSF (S1A and S1B Fig). The cells were slightly positive for Periodic acid–Schiff (PAS) due to the synthesis of glycoproteins (SCO-spondin) in the ER (Fig 1B). After biosynthesis, glycoproteins are concentrated in secretory granules in the apical region of the cells, which showed PAS staining (Fig 1B; inset, arrowheads and S1C Fig). The SCO is delimited by ependymal cells, generated by an abrupt transition from cylindrical to cubical ciliated epithelium (Fig 1B, asterisks). In normoglycemia (3 mM glucose in CSF, control condition), we observed that SCO cells expressed the glucose transporter, GLUT2 (green staining in C), a hexose carrier classically expressed in glucose-sensing cells (e.g., pancreatic β-cells, tanycytes, and hepatocytes), in the apical area of the cells (*N* = 3) (Fig 1C, inset, and 1F) [25]. Additionally, GLUT1 was detected mainly in basal region of SCO cells (Fig 1D and 1F) and in the apical zone of ependymal cells (Fig 1D, inset). GLUT6, a low-affinity glucose transporter, was not observed (Fig 1E and 1F).

These data suggest that SCO cells actively take up glucose; therefore, we injected the fluorescent glucose analog, D-glucose analog 2-[N-(7-nitrobenz-2-oxa-1,3-diazol-4-yl) amino]-2-deoxy-D-glucose (2-NBDG), into the vasculature and found that glucose entered in SCO cells (*N* = 3) (Fig 1G) as well as ventricular ependymal cells (Fig 1H, white arrows). Using a previously validated intraperitoneal (IP) glucose injection protocol [1], glucose in the CSF reached concentrations of 3.2 ± 1.1 mM (after 30 min in CSF isolated from third ventricle, control) (*N* = 25), 5.4 ± 0.5 mM (*N* = 4) (low hyperglycemic conditions), and 10.2 ± 0.6 mM (*N* = 10) (high hyperglycemic conditions). When the CSF glucose concentration was 10 mM, GLUT2 was also expressed in apical regions in SCO cells (Fig 1I, black arrows and inset). Furthermore, SCO cells showed a dissimilar structural pattern, with a “shiny” cytoplasm (Fig 1I) and “band-like” apical structures (Fig 1I, black arrow and inset), when compared to the control condition, where GLUT2 was detected mainly in the apical region of the cells without apparent structural changes (Fig 1J, black arrows and inset). Conversely, GLUT2-negative dorsal ependymal cells did not show any apparent changes (Fig 1I and 1J, ependyma). Furthermore, to rule out ventricular enlargement at CSF glucose concentrations of 10 mM, we performed T2-weighted coronal MRI (*N* = 4), which showed no abnormal expansion of the third ventricle (Fig 1K and 1L).

Next, we studied structural and secretory activity changes in SCO cells under normoglycemia and hyperglycemia. Immunohistochemical analysis with an anti-SCO-spondin antibody revealed that glycoproteins were specifically detected in the cytoplasm (brown staining) (Fig 1M and 1N) (*N* = 4), as previously shown [12]. Additionally, apical blebs, secretory granules and secreted SCO-spondin over the apex of cilia, formed a continuous immunoreactive line (Fig 1N, arrowheads and inset; S1C and S1D Fig). Different cellular changes were observed under hyperglycemic conditions. At a CSF glucose concentration of 5 mM, discontinuous immunoreactivity was observed in the apical regions of SCO cells (Fig 1O, arrowhead), and in the cytoplasm, we observed lower immunoreactivity (*N* = 4) (Fig 1O, arrows). At a CSF glucose concentration of 10 mM, positive immunoreaction in the cytoplasm showed a granular appearance (Fig 1P and 1Q), the intense signal at the apical region observed in the control was not detected, and instead, a “strip-like” edge was observed in the SCO cells (*N* = 4) (Fig 1Q, arrowheads).

To define whether intracellular immunoreactivity positive for anti-SCO-spondin was preferentially associated with the ER, we performed immunofluorescence and confocal microscopy studies with the ER marker, anti-KDEL. Under normoglycemic conditions, SCO-spondin was highly colocalized with anti-KDEL (*N* = 4) (Fig 1R and 1Rˊ). However, the secretory granules detected in the blebs (apical region of the cells) were intensely SCO-spondin positive but KDEL negative (Fig 1R and 1Rˊ, white arrows and inset). When the CSF glucose concentration was increased at 5 mM, anti-spondin immunoreactivity in the ER was decreased, with a slight change in colocalization with KDEL (*N* = 4) (Fig 1S and 1Sˊ; 1U, 1W, and 1X), and the immunoreactivity in the apical zone of the cells was discontinuous and decreased (Fig 1Sˊ and 1V, white arrows and inset). The most evident changes were observed when the CSF glucose concentration was increased at 10 mM CSF. In the SCO cells, ER cisternae exhibited globular structures and decreased immunoreactivity for anti-SCO-spondin, with a slight change in colocalization with KDEL (*N* = 4) (Fig 1T and 1Tˊ; 1U, 1W, and 1X). In addition, the apical region of the cells contained reduced SCO-spondin-positive blebs (Fig 1T and 1Tˊ, white arrows, inset, and 1V). These results suggested that when the CSF glucose concentration increases, SCO-spondin synthesized in the ER is secreted into the CSF, decreasing the positive immunoreactivity in ER, blebs-apical granules, and extracellular zone over the cilia.

**Fig 2:**
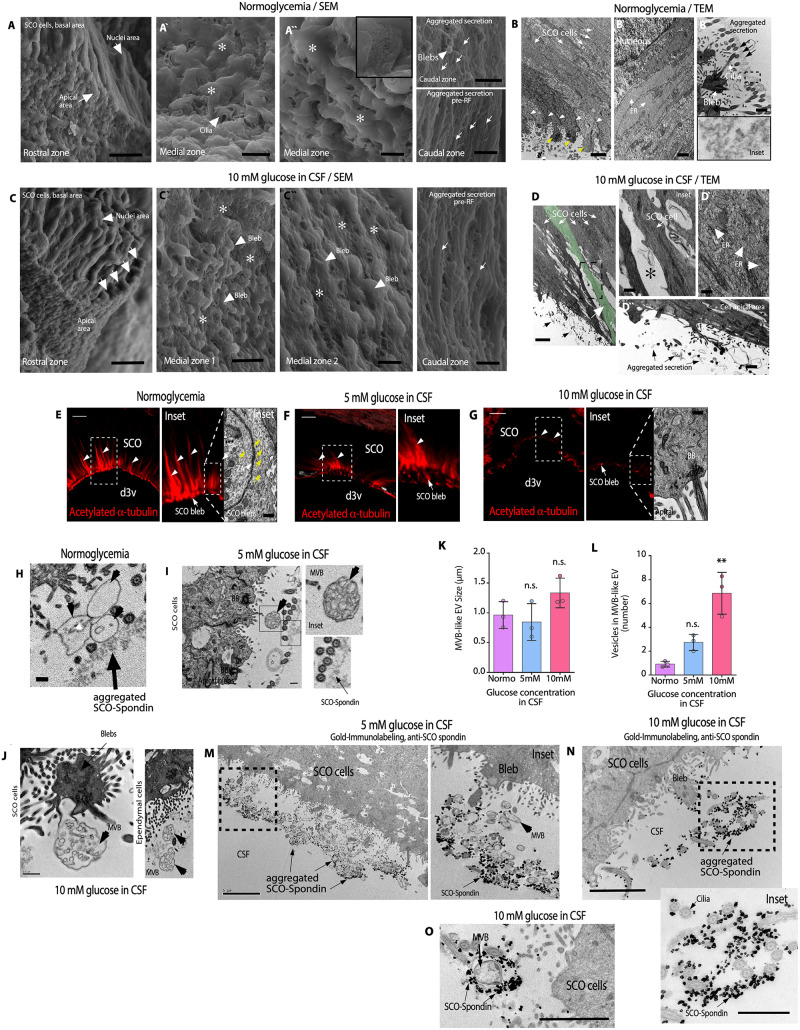
Hyperglycemia changes the normal morphology of SCO cells that secrete SCO-spondin and MVBs-like EV. (**A**) SEM analysis of normoglycemic SCO cells (control) showing basal, nuclear, and apical cell zones. Scale bar: 10 μm. The apical zone was covered by a layer of SCO-spondin secretion (Aˋ and Aˋˋ, asterisks). Scale bar: 2 μm. The thin layer of SCO-spondin presented less compaction in some parts (Aˋˋ, inset). In the caudal region of the SCO, the SCO-spondin presented different degrees of aggregation or it formed pre-RF (A, caudal zone images, white arrows). *N* = 3. Scale bar: 10 μm or 1 μm in pre-RF. (**B**) TEM analysis of normoglycemic animals. SCO cells were elongated (white arrows) and formed an epithelium without intercellular spaces. The cells contained secretory granules inside blebs (yellow arrows) and a junction complex in the apical region (B, white arrowheads). *N* = 5. Scale bar: 2 μm. SCO cells showed elongated ER cisternae full of secretory material (Bˋ, white arrows). Scale bar: 0.5 μm. Apical zone of the cells with secreted SCO-spondin forming a thin protein layer (Bˋˋ, black arrows and inset). Scale bar: 0.5 μm. (**C**) SEM analysis of hyperglycemic SCO cells showing basal, nuclear, and apical cell zones. *N* = 3. Scale bar: 10 μm. The apical zone is covered by scarce SCO-spondin secretion (Cˋ and Cˋˋ, asterisks). In some SCO medial regions, blebs form rounded structures (Cˋ and Cˋˋ, arrowheads). Scale bar: Cˋ, 5 μm; Cˋ, 2 μm. In the caudal zone of the SCO, the SCO-spondin presented different degrees of aggregation (C, caudal zone images, white arrows). Scale bar: 1 μm. (**D**) TEM analysis of hyperglycemic animals. SCO cells were contracted (white arrows and green cell) and had intercellular spaces (inset and asterisk) *N* = 5. Scale bar: D, 2 μm; inset, 1 μm. SCO cells showed fragmented ER cisternae (Dˋ and arrowheads). Scale bar: 0.5 μm. Sparse secretory material was observed extracellularly in contact with the cilia. (Dˋˋ, black arrows). Scale bar: 0.4 μm. (**E-G**) Immunofluorescence and TEM analysis. Changes in acetylated α-tubulin distribution and intensity were observed in the apical region of SCO cells under normoglycemic (E, white arrowheads) and hyperglycemic (F and G, white arrowheads) conditions. *N* = 3. Scale bar: 2 μm. TEM also revealed a reduction in microtubules detection in the apical area of the cells (E, yellow arrows and G). Scale bar: 2 μm. (**H-J**) TEM analysis of apical SCO cells and ependymal cells. MVBs-like EV (arrowheads) were detected extracellularly in samples from normoglycemia and hyperglycemic animals (*N* = 3). Scale bar: H and I, 0.3 μm; J, 0.5 μm. (**K** and **L**) The plots show the size of MVB-like EV (K) or number of vesicles in MVB secreted by SCO cells. The graph showed data from 3 biologically independent samples. The error bars represent the SD. **P < 0.01, n.s. = not significant (one-way ANOVA). (**M-O**) Gold immunolabeling with anti-SCO-spondin and TEM microscopy in SCO cells under hyperglycemic conditions, 5 mM glucose in CSF (M and inset) or 10 mM glucose in CSF (N and inset). MVBs-like EV were observed mixed with SCO-spondin and the apex of cilia (*N* = 3) (O). Scale bar: M, 5 μm; N and O, 2 μm. Inset in N, 1 μm. Data used to generate graphs can be found in S1 Data. BB, basal body; CSF, cerebrospinal fluid; ER, endoplasmic reticulum; MVBs-like EV, multivesicular bodies-like extracellular vesicle cluster; RF, Reissner’s fibers; SCO, subcommissural organ.

We hypothesized that previously observed changes in the apical region of SCO cells could be analyzed using scanning and transmission electron microscopy (SEM and TEM, respectively). In normoglycemic conditions (control), SEM analysis showed that SCO cells are detected forming a compact epithelium (Fig 2A). In SCO rostral and medial zones, the apical blebs with cilia and microvilli are covered by SCO-spondin secretion forming a layer, which confers a smooth appearance to these structures (Fig 2Aˋ and 2Aˋˋ, asterisks). In the SCO caudal regions, SCO-spondin presents different degrees of aggregation and compaction, forming structures known as pre-RF (Fig 2A, caudal zones and arrows) [12]. TEM analysis showed elongated cells without intercellular spaces between them (*N* = 3) (Fig 2B, white arrows), with apical junctions (arrowheads) and prominent blebs (yellow arrows) (Figs 2B and S1B–S1D). ER cisternae were elongated and full of proteins (Fig 2Bˋ, white arrows). Extracellularly, SCO-spondin was observed forming a continuous layer of secretion in close contact with the apex of the cilia (Fig 2Bˋˋ, arrows and inset).

At CSF glucose concentration of 10 mM, SEM analysis showed that the SCO cells were irregular in appearance, with constricted apical areas (Fig 2C, apical white arrows). In rostral and medial SCO, the blebs were devoid of smooth surface secretion. Therefore, cilia and apical microvilli were also observed, among which secreted material with different levels of aggregation was detected (Fig 2Cˋ and 2Cˋˋ, asterisks). In some areas of the medial zone, the blebs were now smaller and more homogeneous in shape (Fig 2Cˋˋ, arrowheads). It cannot be ruled out that we were also observing MVBs (see below), which can be similar in size to the blebs (Fig 2I). In the SCO caudal zone, SCO-spondin was aggregated, adopting the well-known pre-RF structure [12] (Fig 2C, caudal zone image, arrows). TEM analyzes showed that SCO cells were constricted with dilated intercellular spaces (Fig 2D, insets and asterisks; S2A–S2C Fig; and S3A, S3C, and S3D Fig). The ER cisterns were observed fragmented (Fig 2Dˋ, arrowhead) and secreted and aggregated proteins were detected outside SCO cells (*N* = 3) but forming a discontinuous and less homogeneous structure than that observed in the control (Fig 2Dˋˋ, black arrow; S2D and S3D Figs). We have not observed structural changes in choroid plexus cells and posterior commissure in hyperglycemic conditions (S2G and S3B Figs). These data suggest that hyperglycemia generated structural changes in SCO cells, increased secretion of SCO-spondin that remains soluble in CSF, and dispersed partially disaggregated SCO-spondin from the apical surface of the SCO.

In order to characterize some of the subcellular changes induced in the apical zone of the SCO cells, the structure of the microtubules abundantly arranged in this zone of their cells was analyzed, which are involved in the structural maintenance and in vesicular secretion mechanisms [12]. We observed that microtubules (acetylated α-tubulin–positive cells) were prominently distributed in the apical area of cells under control conditions (Fig 2E, white arrowheads and insets), which was confirmed by TEM analysis (Fig 2E, yellow arrows). Changes in fluorescence intensity and distribution of the signal were observed when the CSF glucose concentration increased at 5 mM or 10 mM CSF (Fig 2F and 2G). Surprisingly, acetylated α-tubulin reactivity almost disappeared in the SCO apical region at 10 mM (Fig 2G, insets). The apical microtubule network was not observed under TEM analysis (Fig 2G, inset TEM). We conclude that hyperglycemia generates structural changes in the apical zone of SCO cells, which may suggest an increased secretion activity of the cells.

Finally, detailed ultrastructural analysis by TEM showed the presence of MVBs with extracellular vesicle (EV) clusters (MVBs-like EV) [26,27]. Under normoglycemic and hyperglycemic conditions, MVB-like EV on SCO cells and ependymal cells were detected (Fig 2H, 2I and 2J, arrowheads, and S1E, S2D, and S3E Figs). The size of the small vesicles (exosome-like vesicles) was 50 to 200 nm (*n* = 150, in different conditions). Under normoglycemic conditions and when the CSF glucose concentration was 5 or 10 mM, MVBs with similar sizes were observed but they showed different EV numbers (Fig 2K and 2L) (quantification in TEM images). To 5 mM glucose in CSF, variable groups of MVBs were observed extracellularly in the apical region of the SCO cells. Using gold immunostaining with anti-SCO-spondin and TEM analysis, SCO-spondin was detected intermixed with MVBs (Fig 2M and inset). Similar results were detected using anti-SCO-spondin with gold immunostaining and TEM at 10 mM glucose in CSF; however, a reduced amount of secreted SCO-spondin and MVBs was detected (Fig 2N and inset). Some MVBs were observed completely surrounded by SCO-spondin and in contact with the cilia (Fig 2O). MVB-like EV clusters were not observed in choroid plexus cells (S2G Fig). These data show that hyperglycemia induced structural changes in SCO cells, increased SCO-spondin secretion, and detached SCO-spondin extracellular film, probably allowing the release of MVBs into the ventricular CSF.

### SCO-spondin secretion decreases in AAV-GFAP-Cre-GFP-injected GLUT2loxp/loxp mice under hyperglycemic conditions

**Fig 3:**
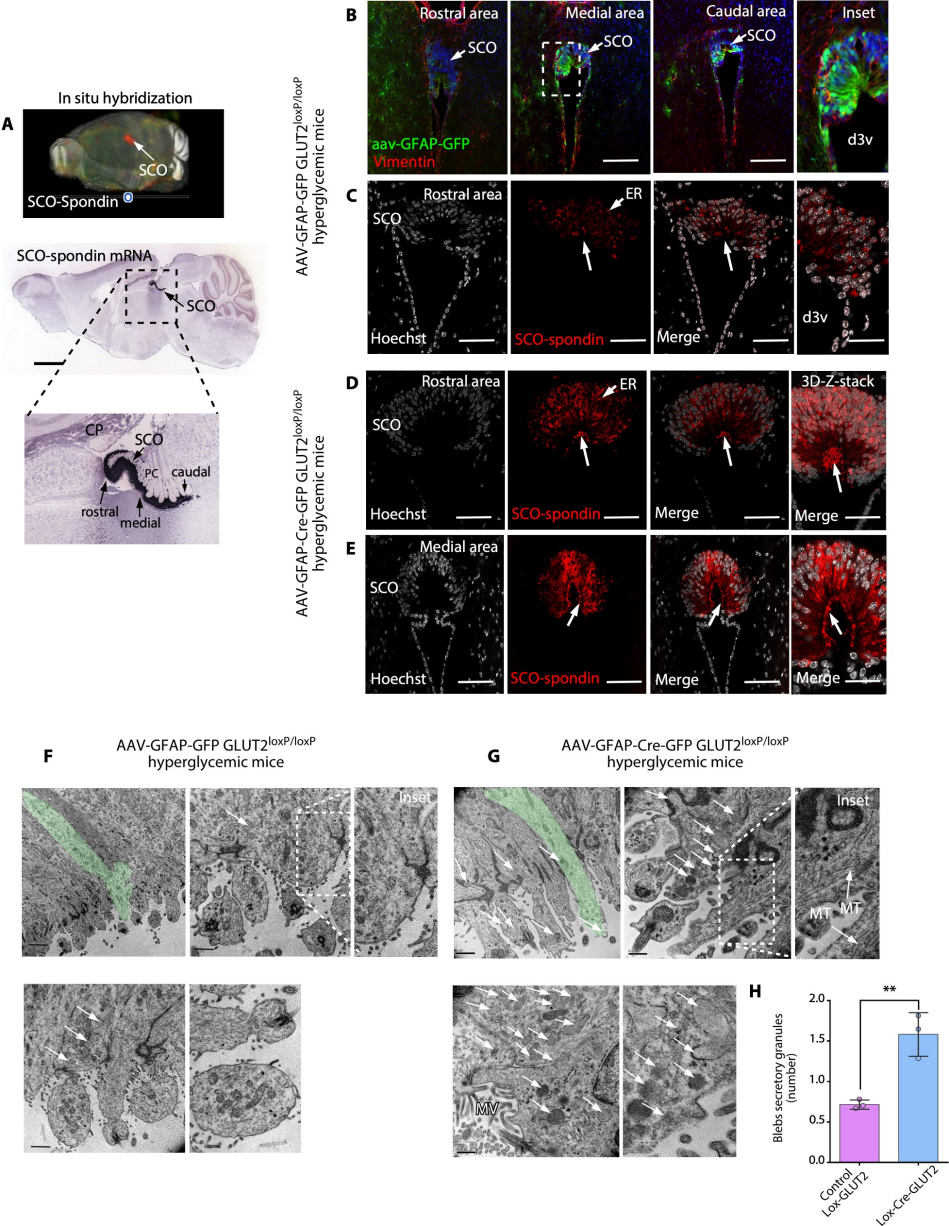
When the CSF glucose concentration is increased, the release of secretory granules by SCO cells is decreased in GLUT2^loxP/loxP^ mice after genetic inactivation of GLUT2. (**A**) 3D brain imaging and sagittal sections after in situ hybridization showing SCO-spondin expression restricted to the commissural area epithelia of the third ventricle in the adult mouse brain. SCO-spondin is shown in red in the 3D brain image, and in blue, in the sagittal sections. Scale bar: 2 mm. *Image credit*: *Allen Institute*. (**B**) Immunohistochemical staining of vimentin in frontal brain sections and analysis of AAV-GFAP-GFP expression in GLUT2^loxp/loxp^ mice. Scale bar: 400 μm. (**C**) SCO-spondin (red) and Hoechst (white; nuclei) staining in AAV-GFAP-GFP–injected hyperglycemic GLUT2^loxp/loxp^ mice. Secretory SCO-spondin was detected in ER cisternae; however, secretory apical granules were not detected (arrow and inset). Scale bar: 200 μm. (**D** and **E**) SCO-Spondin (red) and Hoechst (white; nuclei) staining in AAV-GFAP-Cre-GFP–injected hyperglycemic GLUT2^loxp/loxp^ mice. Secretory SCO-spondin was detected in ER cisternae and secretory apical granules (arrow and inset). Scale bar: 200 μm. (**F** and **G**) TEM analysis of AAV-GFAP-GFP–injected hyperglycemic GLUT2^loxp/loxp^ and AAV-GFAP-Cre-GFP–injected hyperglycemic GLUT2^loxp/loxp^ mice. Elongated SCO cells (green cells) were visualized at low magnification, and apical blebs were visualized at higher magnification. The secretory granules are indicated by white arrows. Scale bar: lower magnification, 2 μm; higher magnification, 0.5 μm. (**H**) Quantification of the number of SCO cell secretory granules in AAV-GFAP-Cre-GFP–injected hyperglycemic GLUT2^loxp/loxp^ mice and AAV-GFAP-GFP–injected hyperglycemic GLUT2^loxp/loxp^ mice. The graph shows data from 3 biologically independent samples. The error bars represent the SD; **P < 0.01 (two-tailed Student *t* test). Data used to generate graph can be found in S1 Data. CSF, cerebrospinal fluid; d3v, dorsal third ventricle; ER, endoplasmic reticulum; GLUT2, glucose transporter 2; MT, microtubule; MV, microvilli; SCO, subcommissural organ.

We first confirmed the expression of SCO-spondin in mice by in situ hybridization, and similar SCO-spondin expression was observed in the rostral, medial, and caudal regions of the SCO (Fig 3A).

To further determine the role of GLUT2 in the activation of SCO cell secretion in response to hyperglycemia, we inactivated GLUT2 by injecting AAV-GFAP-Cre-GFP or AAV-GFAP-GFP into the SCO of GLUT2^loxp/loxp^ mice (control, blood glycemia = 8.6 ± 0.3 mM, *N* = 6) and performed analyses under hyperglycemic conditions. The value of induced hyperglycemia in mice was 18 ± 3 mM (*N* = 6). The virus was adequately expressed in SCO cells in the rostral, medial, and caudal areas of the SCO (Fig 3B). Some ependymal cells also showed a positive signal, indicating GFAP expression. In mice injected with AAV-GFAP-GFP (control, functional GLUT2), SCO-spondin did not accumulate in the apical blebs of SCO cells, and lower SCO-spondin immunoreactivity was detected in the ER and blebs (Fig 3C, white arrow).

These data strongly suggested that SCO-spondin is secreted into the CSF under hyperglycemic conditions. TEM showed that there were few secretory granules in blebs in AAV-GFAP-GFP–injected GLUT2^loxp/loxp^ mice under hyperglycemic conditions (Fig 3F, black arrows). In addition, shorter and rounder blebs with a lower microtubule content were observed (Fig 3F, higher magnification). Interestingly, the cells did not present a contracted morphology, even though their apical secretory granules had been released (Fig 3F, green cell in low magnification), as previously observed for rat cells. In a complementary analysis, we observed that rat and mouse SCO cells showed considerable differences in the levels and distribution of β-catenin, a junction complex marker. While rat SCO cells did not show β-catenin positivity in the intermediate cytoplasmic zone (S4A and S4C Fig), mouse SCO cells showed strong β-catenin immunoreactivity in the cell membrane throughout the cell border, with even more intense staining being observed in the apical and lateral cytoplasmic regions (S4D Fig). This could explain why mouse SCO cells did not shrink when they were induced to secrete their contents under hyperglycemic conditions. Similar results were observed by TEM analysis (S4B, S4E, and S4F Fig).

Conversely, in mice injected with AAV-GFAP-Cre-GFP, which inactivates only GLUT2 function, apical secretory granules accumulated in SCO cells, and SCO cells showed higher SCO-spondin immunoreactivity in the ER and blebs (Fig 3D, white arrow). It was evident that SCO cells were larger and exhibited a larger space between the nucleus (white staining) and the blebs (Fig 3D). Similar results were observed in both the rostral and medial areas of the SCO (Fig 3D and 3E). These data strongly suggested that SCO-spondin is not actively secreted into CSF under hyperglycemic conditions in mice in which GLUT2 is inactivated. TEM revealed that under hyperglycemic conditions, cells with longer blebs and robust microtubular structures were observed (Fig 3G, inset). Furthermore, there were significantly more secretory granules in the apical region in AAV-GFAP-Cre-GFP–injected GLUT2^loxp/loxp^ mice under hyperglycemic conditions (Fig 3G, white arrows and H) (*N* = 3). Therefore, GLUT2 is partially required for SCO-spondin release.

Interestingly, we also observed that GLUT2 was not detectable in 1.5-year-old rats (S5A Fig) and that the cellular features in 1.5-year-old rats with a CSF glucose concentration of 10 mM were very similar to those of normoglycemic animals (controls). Specifically, there were many α-acetylated tubulin-positive signals and secretory granules in apical blebs SCO-positive (S5A Fig, white arrows; S5D Fig, apSGs). Extracellularly, SCO-spondin (S5C Fig, inset), and large empty MVBs (greater than 3 μm) were observed (S5C Fig, inset and arrowheads). Additionally, no cell contraction was observed (S5C and S5D Fig). Finally, no secreted SCO-spondin was detected contacting dorsal ependymal cells (S5B and S5E Fig). In the subependymal area, lucid structures were observed, suggesting parenchymal edema formation (S5E Fig, insets). Therefore, only in old animals, GLUT2 was not expressed and SCO-spondin release did not increase in response to hyperglycemia.

### Hyperglycemia induces changes in SCO cells, stimulating the release of SCO-spondin into the CSF, which interacts with ependymal cell cilia

**Fig 4:**
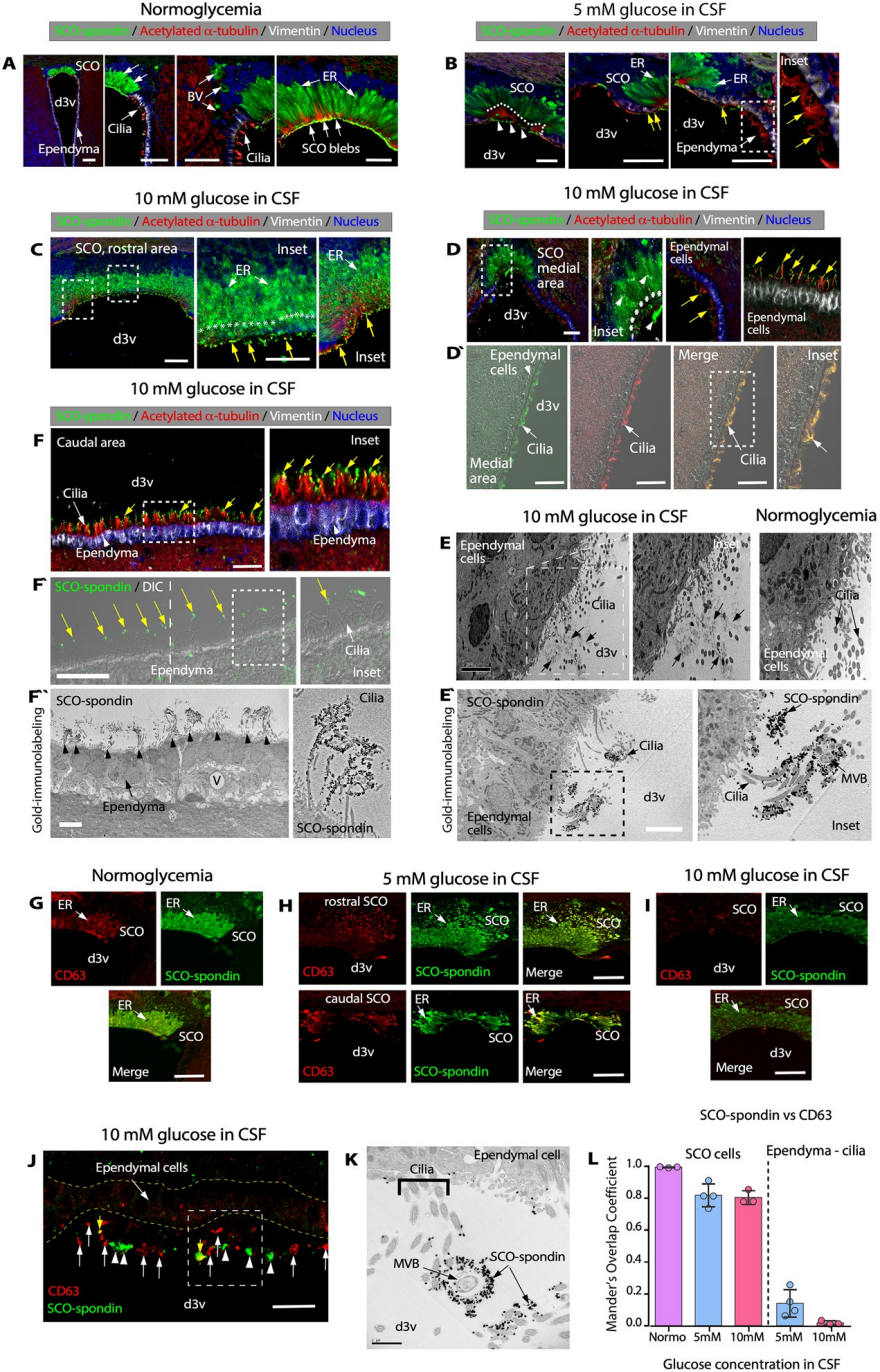
Hyperglycemia increases the secretion of SCO-spondin- and CD63-positive vesicles, which is present at the apex of ependymal cells. (**A-C**) Immunohistochemical staining of SCO-spondin (green), vimentin (white), and acetylated α-tubulin (red) in sections of the SCO under normoglycemic conditions (A) and hyperglycemic conditions (CSF glucose concentration of 5 mM (B) or 10 mM (C)). Nuclear staining with Hoechst (blue). Scale bar: 50 μm. SCO-spondin–positive blebs, ER, BV, and cilia are depicted. The pattern of intracellular SCO-spondin immunoreactivity was altered under different glycemic conditions. (**D** and **D**ˋ) Immunohistochemical staining of SCO-spondin (green), vimentin (white), and acetylated α-tubulin (red) in frontal brain sections from hyperglycemic rats (CSF glucose concentration of 10 mM). Nuclear staining with Hoechst (blue). Scale bar: 30 μm. Most secreted SCO-spondin was detected at the apex of ependymal cells (yellow arrows). Immunofluorescence and DIC (D`) microscopy confirmed that cilia and SCO-spondin interacted. (**E**) TEM revealed the presence of aggregated extracellular proteins at the apex of ependymal cells (arrows) in hyperglycemia. Scale bar: 5 μm. (**Eˋ**) Gold immunolabeling and TEM using anti-SCO-spondin in ependymal cells, caudal area; 10 mM glucose CSF. SCO-spondin–positive reaction (black gold particles) were detected in the cilia. Scale bar: 5 μm. (**F** and **F**ˋ) Immunohistochemical staining of SCO-spondin (green), vimentin (white), and acetylated α-tubulin (red) in frontal brain sections (caudal area) from hyperglycemic rats (CSF glucose concentration of 10 mM). Nuclear staining with Hoechst (blue). Scale bar: 30 μm. Most secreted SCO-spondin was detected at the apex of ependymal cells (yellow arrows). Immunofluorescence and DIC (Fˋ) microscopy confirmed that cilia and SCO-spondin interacted. (**F**ˋˋ) Gold immunolabeling and TEM using anti-SCO-spondin in ependymal cells; medial area. SCO-spondin–positive reaction (black gold particles) was detected in the cilia. Scale bar: 5 μm. (**G-I**) Immunohistochemical staining of SCO-spondin (green) and CD63 (red) in frontal brain sections containing SCO cells under different glycemic conditions. Intensity reactivity was detected for the exosome marker CD63, mainly in ER cisternae. Scale bar: 80 μm. (**J**) Immunohistochemical staining of SCO-spondin (green) and CD63 (red) in sections containing ependymal cells under hyperglycemic conditions. CD63 (large arrows) was detected at the apex of ependymal cells, which its colocalization with SCO-spondin was lower (arrowheads). Scale bar: 30 μm. (**K**) Gold immunostaining with anti-OSC-spondin and TEM analysis in ependymal cells. Scale bar: 2 μm. (**L**) Quantification of Mander’s overlap coefficient for SCO-spondin and CD63 in SCO and ependymal cells under different glycemic conditions. In normoglycemia, CD63 and SCO-spondin are not detected in ependymal cells. The graph shows data from 4 biologically independent samples. The error bars represent the SD. Data used to generate graph can be found in S1 Data. (D) SCO-spondin immunoreactivity was also observed in SCO cells and pre-RF (Fig 4D, inset and arrowheads), and positive immunoreaction was detected close to the apex of ependyma cilia (Fig 4D, yellow arrows). Using DIC analysis in ependymal cells (Dˋ), we also observed positive immunoreaction for SCO-spondin and acetylated α-tubulin on the ependymal cilia (Fig 4Dˋ, white arrows). TEM analysis showed that aggregated proteins were present in the apex of ependymal cells cilia at 10 mM glucose in CSF (Fig 4E, arrows). These secreted materials were not observed in the control ependymal cells (Fig 4E, normoglycemia). Gold immunostaining with anti-SCO-spondin and TEM analysis confirmed SCO-spondin associated with the apex of ependymal cells and MVBs (Fig 4E’and inset; S3F and S3G Fig). BV, blood vessels; DIC, differential interference contrast; d3v, dorsal third ventricle; ER, endoplasmic reticulum; MVB, multivesicular body; SCO, subcommissural organ.

As mentioned above, under normoglycemic conditions, SCO-spondin was localized in the ER and blebs (Fig 4A). When the CSF glucose concentration was increased (5 mM), evident changes in the apical region of SCO cells were observed; SCO-spondin–positive blebs were present (B, arrowheads), and ER immunoreactivity was decreased (Fig 4B, white arrows). We also detected released SCO-spondin on ependymal cells (Fig 4B, yellow arrows and inset; S2E and S2F Fig). The most evident changes were observed when the CSF glucose concentration was increased to 10 mM CSF. In the SCO, ER cisternae exhibited a globular structure (Fig 4C, white arrows and insets). In addition, the apical region of the cells did not contain SCO-spondin–positive blebs, only disaggregated secretion (Fig 4C, yellow arrows), suggesting that many of the apical secretory granules and extracellular aggregated SCO-spondin were released into the CSF under hyperglycemic conditions. Interestingly, in the medial SCO.

Similar results were observed in the dorsal third ventricle, where SCO-spondin was also detected intermingled with the cilia of the ependymal cells (Fig 4F, yellow arrows and inset). The interaction between cilia (white arrow) and SCO-spondin (yellow arrows) was also detected using differential interference contrast (DIC) analysis (Fig 4Eˋ, yellow arrows and inset) and by using gold immunostaining with anti-SCO-spondin and TEM analysis, which demonstrated that SCO-spondin interacts with ependymal cells cilia (Fig 4Fˋˋ, arrowheads and cilia high magnification image). We concluded that soluble SCO-spondin is gradually released into the CSF at different glucose levels and that the secreted SCO-spondin reaches the apices of ependymal cell cilia.

We also observed that SCO cells were positive for the exosome marker, CD63, which was highly colocalized with SCO-spondin, mainly in basal ER cisternae (Fig 4G–4I and 4K). At a CSF glucose concentration of 10 mM, CD63 immunoreactivity was decreased, mainly in perinuclear ER cisternae (Fig 4I), suggesting increased vesicle secretion. CD63 was also detected on ependymal cells only under hyperglycemia, where poor colocalization between CD63 and SCO-spondin was observed (Fig 4J, arrowheads for SCO-spondin, white arrows for CD63, yellow arrows for colocalization). Colocalization analysis of CD63 and SCO-spondin (*N* = 4) showed high colocalization in SCO cells but poor colocalization in ependymal cells (Fig 4L) when the CSF glucose concentration was 5 mM or 10 mM. Only some focal areas showed colocalization between CD63 and SCO-spondin, confirming what was observed by gold immunostaining and TEM in ependymal cells, closer to the SCO (Fig 4J, yellow arrows and 4K). Then, the secretion of SCO-spondin- and CD63-positive vesicles were increased in hyperglycemia, which was detected in the apex of ependymal cells.

### The SCO-RF complex is not structurally altered under hyperglycemic conditions, but SCO-spondin release into the CSF is associated with ependymal cell cilia

**Fig 5:**
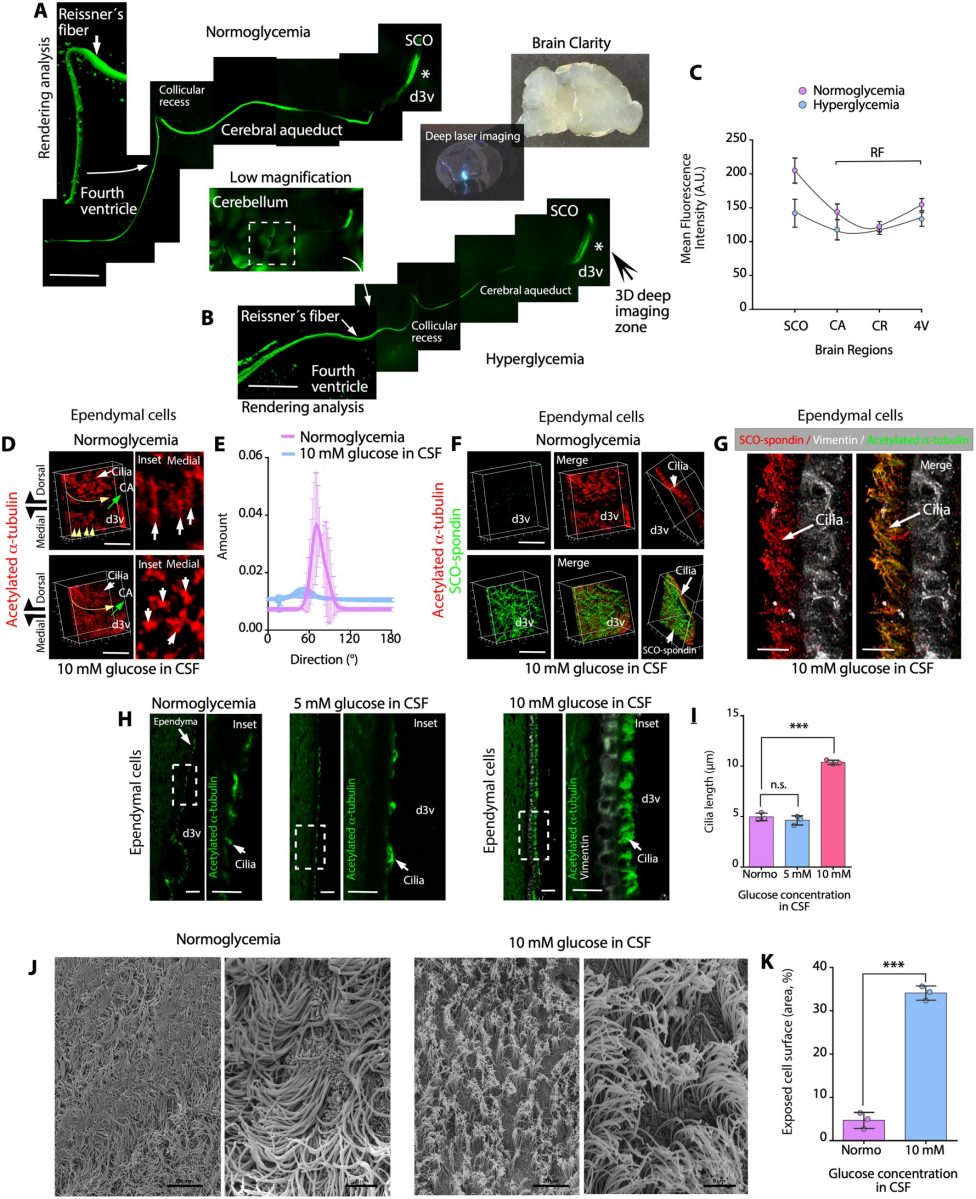
SCO-spondin interacts with ependymal cell cilia, increasing cilia stiffness. (**A, B**) Immunohistochemical staining of SCO-spondin in brain slices from normoglycemic and hyperglycemic animals following the CLARITY protocol. The SCO was detected in the d3v and RFs along the CA, CR, and 4V. Scale bar: 200 μm. (**C**) Quantitative analysis of SCO-spondin immunohistochemical staining under different glycemic conditions. The mean fluorescence intensity was analyzed in SCO and RF observed in CA, CR, and 4V. The graph shows data of 3 biologically independent samples. The error bars represent the SD. (**D, E**) 3D deep CLARITY confocal analysis after immunohistochemical staining of acetylated α-tubulin in the d3v (the asterisk in B indicates the area analyzed) under normoglycemic and hyperglycemic conditions. The ciliary orientation is indicated by the yellow arrows (curved or straight). The posterior localization of the CA is indicated by the green arrow. Scale bar: 200 μm. The orientation of the ciliary structures in different ependymal cells from medial region are observed in the insets (D, white arrows) and in the directionality histogram that represents the number of objects with a specific ciliary orientation pattern (^o^), under normoglycemic and hyperglycemic conditions. The graph shows data from 3 biologically independent samples. (**F**) 3D deep CLARITY confocal analysis after immunohistochemical staining of SCO-spondin (green) and acetylated α-tubulin (red) under normoglycemic and hyperglycemic conditions. At a CSF glucose concentration of 10 mM, SCO-spondin was detected at the apex of ciliary structures. Scale bar: 200 μm. (**G**) Immunohistochemical staining of SCO-spondin (red), vimentin (white), and acetylated α-tubulin (green) in ependymal cells in the brains of hyperglycemic animals and confocal superresolution-Lightning/z-stack analysis. SCO-spondin was observed as a particulate secretion attached to cilia. Scale bar: 10 μm. (**H**) Immunohistochemical staining of acetylated α-tubulin (green) and vimentin (white) in sagittal sections of the brains. Under hyperglycemic conditions (mainly a CSF glucose concentration of 10 mM), cilia were straight, suggesting increased stiffness. Scale bar: 30 μm. (**I**) Quantitative analysis of cilia length in ependymal cells under normoglycemic and hyperglycemic conditions. The graph shows data from 3 biologically independent samples. The error bars represent the SD; ***P < 0.001, n.s. = not significant (one-way ANOVA). (**J**) Scanning electron microscopy of the dorsal third ventricle ependymal cells under normoglycemic and hyperglycemic conditions. Scale bar: 20 μm and 5 μm. (**K**) Exposed apical surface of ependymal cells under normoglycemic or hyperglycemic conditions. The graph shows the area percentage of exposed cell surface; data from 3 biologically independent samples. The error bars represent the SD; ***P < 0.001 (two-tailed Student *t* test). Data used to generate graphs can be found in S1 Data. CA, cerebral aqueduct; CR, collicular recess; d3v, dorsal third ventricle; RF, Reissner’s fibers; SCO, subcommissural organ; 4V, fourth ventricle.

To determine whether hyperglycemia alters the SCO-RF complex, we performed 3D confocal analysis of thick brain sections (2 mm) after performing the CLARITY protocol (Fig 5A and 5B) and immunohistochemical analysis with anti-SCO-spondin and anti-acetylated α-tubulin antibodies. Under normoglycemic and hyperglycemic conditions, the SCO-RF complex was unaltered, which was detected through the cerebral aqueduct (CA), collicular recess (CR), and the posterior part of the fourth ventricle (4V) (Fig 5A, 5B, and 5C). In some areas, RFs were reconstructed by Z-stack confocal imaging and rendering analysis, and the volume of the RF surface was observed (Fig 5A and 5B, fourth ventricle). In a second analysis, we performed 3D reconstruction of the dorsal and medial walls of the third ventricle ependymal cells. Z-stack analysis was performed for ependymal cells near the frontal SCO region (Fig 5B, asterisk), covering a distance of 1.4 mm from the dorsal to medial area of third ventricle (Fig 5D). In the dorsal area, a curved pattern of acetylated α-tubulin–positive cilia (the hypothetical fluid flow is indicated by the curved yellow line) was generated by the curved wall of the thalamus (Fig 5D, dorsal), which pushes the CSF into the CA under normoglycemic and hyperglycemic conditions (green arrow). In the medial normoglycemic third ventricle (Fig 5D, medial and inset, white arrows), a rectilinear pattern was observed for acetylated α-tubulin–positive cilia (the hypothetical fluid flow is indicated by the yellow arrows); however, under hyperglycemic conditions, acetylated α-tubulin staining exhibited focal reactivity (Fig 5D, medial and inset, white arrows). The orientation degree of the ciliary structures in the normoglycemic condition was 74 ± 8°. On the other hand, in the hyperglycemic condition, it was 39 ± 20° (Fig 5E) (*N* = 3). This analysis suggests that the cilia in the medial region of the ventricle has different orientation patterns in the diverse experimental conditions.

We also analyzed SCO-spondin reactivity under normoglycemic conditions and found low-to-absent immunoreactivity on the ependymal surface (Fig 5F). In parallel, we analyzed SCO-spondin reactivity under hyperglycemic conditions and found that it formed “waves” widely distributed on ependymal surface (Fig 5F). In general, the colocalization between SCO-spondin and acetylated α-tubulin was not 100%. Superresolution lightning microscopy revealed that under hyperglycemic conditions, SCO-spondin interacted with the external region or surface of cilia, in the dorsal ependymal wall, forming small aggregates that were distributed along its structure (Fig 5G, arrows).

The lengths of the ciliary structures were substantially different under normoglycemic conditions, when the CSF glucose concentration was 5 mM and when the CSF glucose concentration was 10 mM (Fig 5H and insets). Under normoglycemic conditions and when the CSF glucose concentration was 5 mM, acetylated α-tubulin immunoreactivity in the cilia alternated between positive and negative zones on the ependymal cell surface (Fig 5H, insets), and the average cilia length was 4.99 ± 0.4 μm and 4.65 ± 0.39 μm, respectively (Fig 5I). However, when the CSF glucose concentration was 10 mM, acetylated α-tubulin immunoreactivity in the cilia showed a continuous positive reaction in the apical zone of the ependymal cells (Fig 5H and inset), and the average cilia length was 10.4 ± 0.91 μm (Fig 5G). Finally, differences in the angular orientation of the cilia in normoglycemia and hyperglycemia were confirmed using scanning electron microscopy of the dorsal third ventricle ependymal wall (Figs 5J and S6A and S6B). In hyperglycemia, the cilia appear rigid and perpendicular to the surface of the ependymal cell. In this position, they left the apical surface of the ependyma more exposed (Fig 5J and 5K) when compared with normoglycemia, a condition that holds the cilia at a more flattened angle for the classic appearance that is actively beating. These data suggested that hyperglycemia and SCO-spondin secretion temporarily alter the ciliary beating angle and the CSF fluid.

### The speed of ependymal flow is significantly slower when the CSF glucose concentration is 10 mM and upon coincubation with SCO-spondin

**Fig 6:**
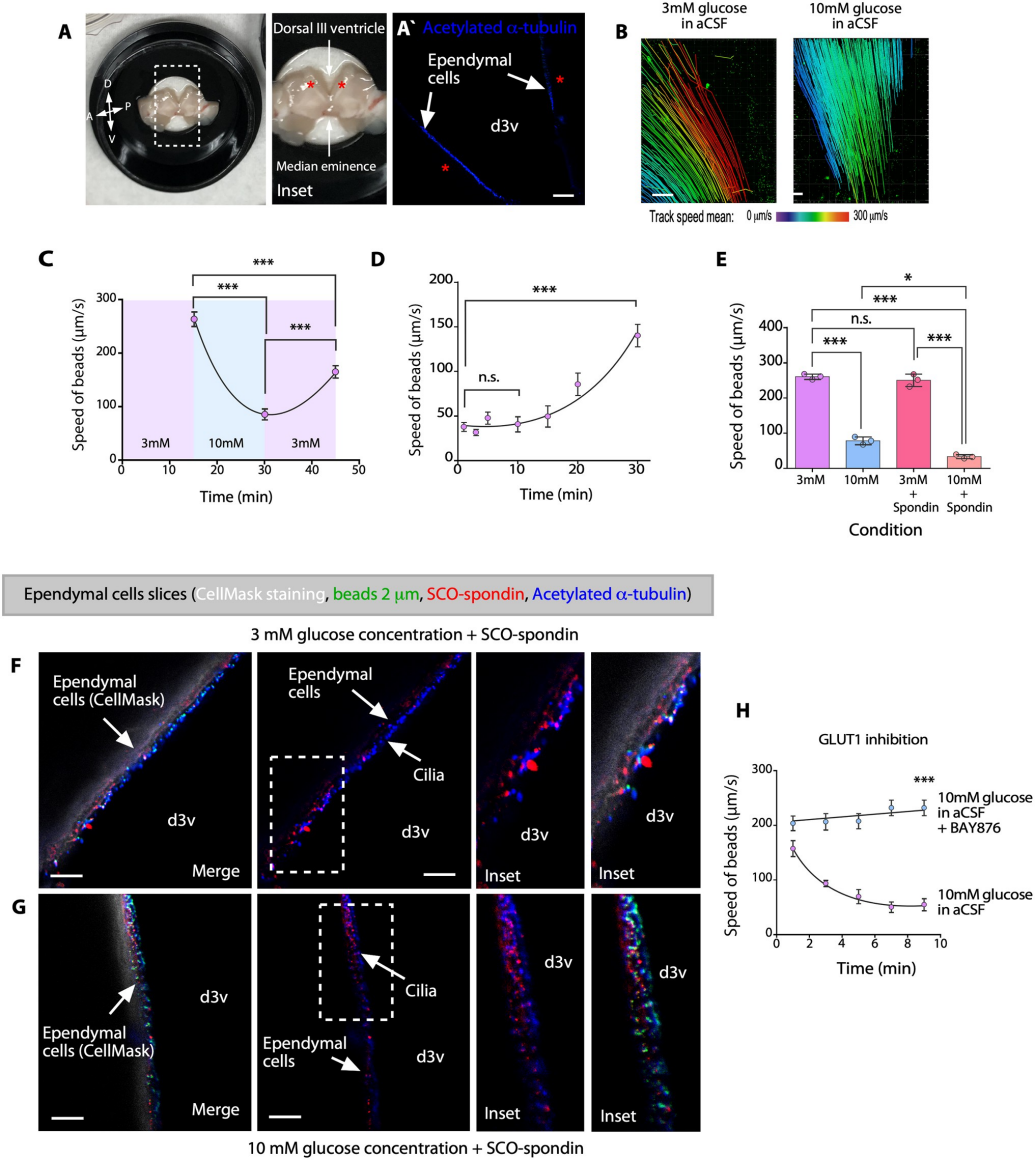
Ependymal flow is significantly slower at high glucose concentrations. (**A, B**) Analysis of the migration speed of fluorescent beads placed on whole-mount preparations of the dorsal wall of the third ventricle (A and inset; A, anterior; D, dorsal; P, posterior; V, ventral.). The fluorescent beads were taken up with a glass needle and placed on whole-mount preparations in the dorsal area (asterisk). Immunofluorescence analysis using anti-acetylated α-tubulin to localize the cilia of ependymal cells (blue signal), in the area used for flow analyses (Aˋ). The speed of fluorescent beads was determined with Imaris software (B). (**C**) The migration speed of the fluorescent beads in aCSF containing 3 mM glucose (control), aCSF containing 10 mM glucose, and aCSF containing 3 mM glucose (recovery). The data are expressed as the mean ± SD. Graph of representative data from 4 independent experiments (***P < 0.001, one-way ANOVA with Tukey’s multiple comparison test). (**D**) Recovery of the speed of the fluorescent beads placed on whole-mount preparations of the dorsal wall of the third ventricle in aCSF containing 3 mM glucose after incubation with aCSF containing 10 mM glucose + bovine SCO-spondin (20 μg/mL) for 15 min. The data are expressed as the mean ± SD. Graph of representative data from 4 independent experiments (***P < 0.001, n.s. = not significant, one-way ANOVA with Tukey’s multiple comparison test). (**E**) Migration speed of the fluorescent beads in aCSF containing 3 mM glucose (same data showed in C), aCSF containing 10 mM glucose (same data showed in C), aCSF containing 3 mM glucose with bovine SCO-spondin (20 μg/mL), or aCSF containing 10 mM glucose with bovine SCO-spondin (20 μg/mL). The data are expressed as the mean ± SD. Graph of representative data from 3 independent experiments (*P < 0.05, ***P < 0.001, n.s. = not significant, one-way ANOVA with Tukey’s multiple comparison test). (**F, G**) Immunohistochemical analysis of whole-mount preparations in 3 mM glucose (control) or 10 mM glucose. CellMask staining (white) and staining of fluorescent microbeads (green), acetylated α-tubulin (1:2,000, blue), and SCO-spondin (1:1,000, red). Scale bar: 10 μm. (**H**) The migration speed of the fluorescent beads that were coincubated or not with aCSF containing 10 mM glucose + BAY876 (GLUT1 inhibitor) after incubation with aCSF containing 3 mM glucose in aCSF containing 10 mM glucose. The data are expressed as the mean ± SD. Graph of representative data from 4independent experiments (***P < 0.001, unpaired *t* test with Welch’s correction). Data used to generate graphs can be found in S1 Data. aCSF, artificial CSF; d3v, dorsal third ventricle; SCO, subcommissural organ.

Abnormalities in cilia beating frequently result in reduced ependymal flow speed [28–31]. Continuous beating of cilia on the apical surface of ependymal cells generates unidirectional fluid flow [32], which can be visualized and quantified by placing polystyrene latex fluorescent microbeads on live preparations of the dorsal/lateral wall of the third ventricle [30,33–35]. We next investigated ependymal flow in whole-mount preparations (Fig 6A), positive for acetylate α-tubulin (Fig 6Aˋ), and incubated with 3 mM glucose-artificial CSF (aCSF) as basal condition, or with 10 mM glucose-aCSF for 15 min (Fig 6B). When a small number of microbeads were placed in the ventral region of the third ventricle (3 mM glucose-aCSF, control), a strong ventro-dorsal to anteroposterior current was observed (Fig 6B). The overall directionality of flow in whole-mount preparations incubated with 10 mM glucose-aCSF was similar to that in control preparations (Fig 6B), but the speed of the fluorescent beads was significantly slower than that in control preparations (Fig 6B). The speed of the fluorescent beads was 261.4 ± 14.1 μm/s after incubation with 3 mM glucose-aCSF for 15 min (control) and was reduced to 78.22 ± 10.8 μm/s after incubation with 10 mM glucose-aCSF after 15 min (Fig 6C). When the samples were reincubated with 3 mM glucose-aCSF, substantial recovery of fluid flow was observed after a short time, with the flow speed reaching 159.67 ± 26.68 μm/s after 15 min (Fig 6C). Additionally, we incubated the samples with 10 mM glucose-aCSF containing 20 μg/mL SCO-spondin for 15 min, and the speed of the beads slowly recovered following incubation with 3 mM glucose-aCSF (Fig 6D). After incubation for 10 to 15 min, no recovery of flow speed was observed; however, after incubation with 3 mM glucose-aCSF for 30 min, the speed of the beads was 140.48 ± 12.72 μm/s (Fig 6D). This finding suggested that SCO-spondin retards the recovery of ciliary movement.

We further compared the data showed in C (3 mM and 10 mM glucose in aCSF), with samples treated additionally with SCO-spondin. In these conditions, the effect was increased when the samples were incubated with 10 mM glucose-aCSF + SCO-spondin, with the flow speed reaching 33.19 ± 5.7 μm/s (Fig 6E, pink analysis). These data demonstrated that SCO-spondin affects ciliary movement under hyperglycemic conditions. To confirm the interaction between SCO-spondin and the ependymal wall in the whole-mount preparations, the ventricular wall was fixed with paraformaldehyde and incubated with CellMask (plasma membrane stain for in vivo analysis), following treatment with glucose-aCSF to identify the ependymal cell membrane (Fig 6F and 6G, white staining). We detected SCO-spondin (red staining) on the surface of ependymal cells in contact with cilia (blue staining) and fluorescent microbeads (green staining) (Fig 6F and 6G). Finally, to determine whether this effect is mediated by the entry of glucose into ependymal cells, we incubated whole-mount preparations in 3 mM glucose for 15 min and subsequently followed by 10 mM glucose (control) or 10 mM glucose containing BAY876, a specific inhibitor of GLUT1 (Fig 6H). No decrease in flow speed was observed within 9 min during this treatment protocol; however, in the control group, the flow speed decreased rapidly from 225.1 ± 16.3 μm/s to 54.72 ± 10.9 μm/s within 9 min. These results indicated that an increase in the glucose concentration decreases ependymal flow and that this effect is enhanced by SCO-spondin.

### SCO-spondin, WNT5a, ROR2, and glypican or testican may form a complex in the apical membrane of ependymal cells

**Fig 7:**
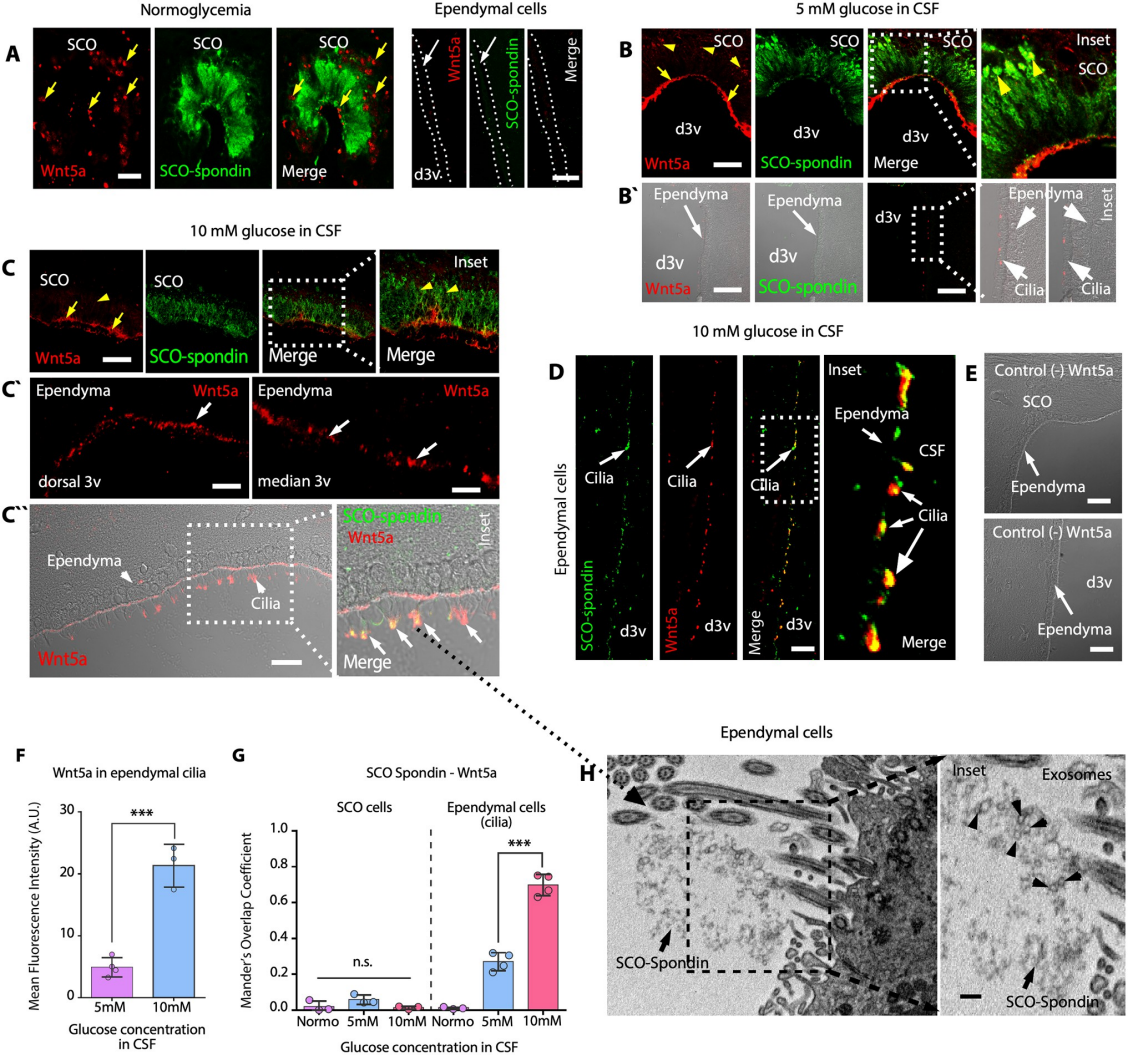
Under hyperglycemic conditions, SCO cells secrete WNT5a, which interacts with ependymal cell cilia. (**A**) Immunohistochemical staining of Wnt5a (red) and SCO-Spondin (green) in frontal brain sections from normoglycemic animals. Focal Wnt5a immunoreactivity was detected most in the basal area of the cells. *N* = 3. Ependymal cells were negative for Wnt5a and SCO-spondin. Scale bar: 20 μm. (**B**) Immunohistochemical staining of Wnt5a (red) and SCO-spondin (green) in frontal brain sections from hyperglycemic animals (CSF glucose concentration of 5 mM glucose). Wnt5a immunoreactivity was mainly observed in the apical part of the cells (yellow arrows). *N* = 3. Immunoreactivity was also observed by DIC microscopy (Bˋ). Ependymal cell cilia were positive for Wnt5a and very weakly positive for SCO-spondin (B`). Scale bar: 20 μm. (**C**) Immunohistochemical staining of Wnt5a (red) and SCO-spondin (green) in frontal brain sections from hyperglycemic animals (CSF glucose concentration of 10 mM). Wnt5a immunoreactivity was observed mainly in the apical area of the cells. *N* = 3. Ependymal cell cilia were strongly positive for Wnt5a (Cˋ). Immunoreactivity was also observed by DIC microscopy. Ependymal cell cilia were strongly positive for Wnt5a and SCO-spondin (Cˋˋ). Scale bar: 20 μm. (**D**) Immunofluorescence staining of SCO-spondin and Wnt5a in ependymal cells cilia. Increased colocalization was observed (white arrows). Scale bar: 20 μm. (**E**) No immunoreactivity was detected when the primary antibody was omitted. Scale bar: 20 μm. (**F**) Quantitative analysis of Wnt5a immunoreactivity under different glycemic conditions. The graph shows data from 4 biologically independent samples. The error bars represent the SD; ***P < 0.001 (two-tailed Student *t* test). (**G**) Quantification of Mander’s overlap coefficient for SCO-spondin and Wnt5a in SCO cells and ependymal cell cilia under different glycemic conditions. The graph shows data from 4 biologically independent samples (Normo in ependymal cells, *N* = 3). The error bars represent the SD; ***P < 0.001, n.s. = not significant (two-tailed Student *t* test). (**H**) TEM analysis. Ependymal cells with aggregated secretions in the apex of cilia. Exosome-like vesicles were also detected (arrowheads and inset). Scale bars: H, 0.3 μm; I, inset, 0.08 μm. Data used to generate graphs can be found in S1 Data. CSF, cerebrospinal fluid; DIC, differential interference contrast; d3v, dorsal third ventricle; SCO, subcommissural organ; TEM, transmission electron microscopy.

It has been shown that secretion of WNT5a stimulates ependymal cell coupling and ciliary beating [36]. We hypothesized that secretion of WNT5a and its interaction with SCO-spondin/heparan sulfate proteoglycan (HSPG)/ROR2 on ependymal cells may enhance the recovery of ciliary movement once glucose levels are normalized. Additionally, it was previously shown that circumventricular organs may produce WNT5a to be transported via lipoprotein particles in the CSF [37]. Using a specific WNT5a antibody validated in Wnt5a−/− mice [37], we found that the WNT5a protein was expressed in the basal and apical regions of columnar cells in the SCO under normoglycemic conditions (Fig 7A, yellow arrows); however, most ependymal cells in the dorsal third ventricle did not express WNT5a (Fig 7A, white arrows). When the CSF glucose concentration was 5 or 10 mM, WNT5a was detected in the apical regions of SCO cells (Fig 7B and 7C, yellow arrows); the weakest immunoreactivity was detected in the ER (Fig 7B, yellow arrowheads and inset), where there was poor colocalization between WNT5a and SCO-spondin (Fig 7B and 7C, yellow arrowheads and inset; Fig 7G). In addition, WNT5a contacted neighboring ependymal cell cilia in the dorsal third ventricle (Fig 7Bˋ, DIC imaging; and Fig 7F). When the CSF glucose concentration was 10 mM, WNT5a was mainly detected in the apical areas of SCO cells (Fig 7C, yellow arrows and inset). Furthermore, WNT5a was broadly detected in the apical area of ependymal cells (Fig 7Cˋ, white arrows), which was confirmed using immunofluorescence with DIC analysis (Fig 7Cˋˋ and 7F) and immunoperoxidase staining (S7A Fig). SCO-spondin colocalized with WNT5a on the cilia of ependymal cells (Fig 7Cˋˋ, 7D, and 7G, white arrows and inset; S7B Fig). When WNT5a primary antibody was excluded, no immunoreactivity was observed in SCO or cilia from ependymal cells (Fig 7E). In contrast, WNT5a immunoreactivity was not detected in tanycytes, lateral ventricle ependymal cells, or choroid plexus epithelial cells (S7A Fig). Under hyperglycemic conditions, disaggregated SCO-spondin detected by TEM was intermingled with ependymal cells cilia, where we also observed an important set of exosome-like vesicles (Fig 7H, arrowheads and inset), which may have contained Wnt5a.

Because WNT5a induces noncanonical β-catenin signaling [38], we analyzed the expression and distribution of ROR2, a transmembrane protein that interacts with WNT5a [38,39]. This receptor was not detected in SCO cells but was observed mainly in dorsal ependymal cells under normoglycemic conditions (S8A Fig, fluorescence and DIC imaging). Diffuse/weak reactivity and some focal reactions near the nucleus were observed under normoglycemic conditions and when the CSF glucose concentration was 5 mM (S8A and S8B Fig). However, when the CSF glucose concentration was 10 mM, ROR2 (eventually clustered) moved to the cell membrane, as indicated by DIC imaging and Z-stack confocal analysis (S8C Fig). These data suggested that the localization of ROR2 in ependymal cells changes at different CSF glucose concentrations.

It has been described that R-spondin family proteins (which contain TSRs similar to those in SCO-spondin) interact with HSPGs to potentiate WNT signaling [40]. Additionally, RORs are associated with HSPG and form a complex to activate signaling [38]. Therefore, we analyzed the expression and distribution of aggrecan, glypican, syndecan, and testican, all of which are HSPGs that, according to the Protein Atlas, can be expressed in ependymal cells. In parallel, we analyzed the distribution of heparan sulfate-6-sulfotransferase 1 (HS6ST1), which was expressed in ependymal cells but not SCO cells under the same experimental conditions (S9A Fig). Aggrecan and syndecan were not expressed in ependymal cells (S9A Fig). Although testican and glypican were not expressed in SCO cells (S9B Fig), both HSPGs were expressed at the apical area in ependymal cells (S9B and S9E Fig). Testican showed higher localization in the apical membrane of ependymal cells; however, similar to ROR2, glypican was observed in clusters (S9B and S9E Fig). The cellular distribution of the studied HSPGs was not obviously different under the different experimental conditions (S9C, S9D, S9F, and S9G Fig). In conclusion, glypican and/or testican are expressed in ependymal cells and may interact with ROR2 to enhance Wnt5a signaling.

### Expression and dynamic distribution of Frizzled-2 in ependymal cells under hyperglycemic conditions

**Fig 8:**
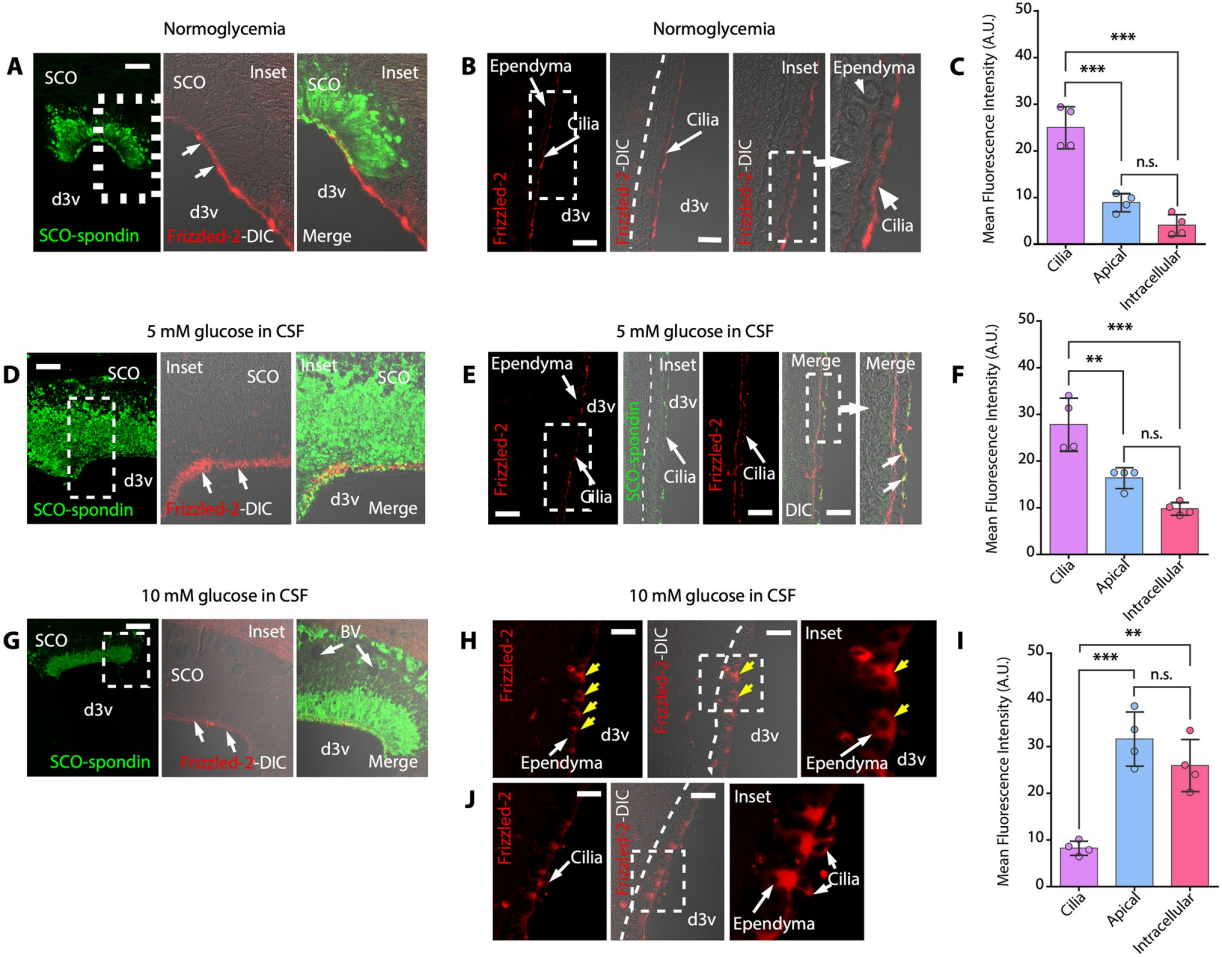
Frizzled-2 is expressed in ependymal cells and is internalized under hyperglycemic conditions. (**A-C**) Immunofluorescence and confocal analysis and/or DIC imaging of the SCO (A) and dorsal ependymal cells (B). Staining of Frizzled-2 and SCO-spondin under normoglycemic conditions. Frizzled-2–positive immunoreactivity was mainly detected in the apex of ciliated cells (B). Scale bar: 20 μm. Quantitative analysis of Frizzled-2 immunoreactivity (C). The graph shows data from 4 biologically independent samples. The error bars represent the SD; ***P < 0.001, n.s. = not significant (one-way ANOVA with post hoc Tukey’s test). (**D-F**) Immunofluorescence and confocal analysis and/or DIC imaging of the SCO (D) and dorsal ependymal cells (E). Staining of Frizzled-2 and SCO-spondin under hyperglycemic conditions (CSF glucose concentration of 5 mM). Frizzled-2 immunoreactivity was detected in the apex of ciliated cells and on the apical membrane of ependymal cells (E). SCO-spondin and Frizzled-2 showed colocalization in cilia (E, DIC image, arrows). Scale bar: 20 μm. Quantitative analysis of Frizzled-2 immunoreactivity (F). The graph shows data from 4 biologically independent samples. The error bars represent the SD; **P < 0.01, ***P < 0.001, n.s. = not significant (one-way ANOVA with post hoc Tukey’s test). (**G-J**) Immunofluorescence and confocal analysis and/or DIC imaging of the SCO (G) and dorsal ependymal cells (H and J). Staining of Frizzled-2 and SCO-spondin under hyperglycemic conditions (CSF glucose concentration of 10 mM). Frizzled-2 immunoreactivity was detected mainly inside the cells (H and J, yellow arrows). Only a few cells showed immunoreactivity in the cilia and cytoplasm (J). Scale bar: 20 μm. (**I**) Quantitative analysis of Frizzled-2 immunoreactivity. The graph shows data from 4 biologically independent samples. The error bars represent the SD; **P < 0.01, ***P < 0.001, n.s. = not significant (one-way ANOVA with post hoc Tukey’s test). Scale bar: 20 μm. Data used to generate graphs can be found in S1 Data. BV, blood vessel; CSF, cerebrospinal fluid; DIC, differential interference contrast; d3v, dorsal third ventricle; SCO, subcommissural organ.

Our results showed that SCO cells express GLUT2, sense high levels of glucose, and secrete SCO-spondin and WNT5a, both of which bind to the surface of ependymal cells, probably through interactions with glypican or testican. Because WNT5a can interact with ROR2 and Frizzled-2 to induce noncanonical β-catenin signaling [38], we analyzed Frizzled-2 expression and distribution. Under normoglycemic conditions, Frizzled-2 was detected in the apical region of SCO cells (*N* = 4) (Fig 8A, arrows). Additionally, Frizzled-2 was mostly detected on cilia in ependymal cells (Fig 8B and 8C). Surprisingly, when the CSF glucose concentration was 5 mM, Frizzled-2 immunoreactivity was present in the apical regions of SCO cells (Fig 8D, arrows) and also observed on cilia and ependymal cells apical membrane (*N* = 4) (Fig 8E and 8F). Colocalization between Frizzled-2 and SCO-spondin in cilia was also detected (Fig 8E, DIC images, arrows). When the CSF glucose concentration was 10 mM, weak Frizzled-2 immunoreactivity was detected in SCO cells (Fig 8G, arrows); however, in ependymal cells, strong cytoplasmatic immunoreactivity was primarily detected (*N* = 4) (Fig 8H, yellow arrows; Fig 8J). Frizzled-2 immunoreactivity was only observed in cilia on a few ependymal cells (Fig 8J, inset). Our data showed that the localization of Frizzled-2 in ependymal cells is modified at different CSF glucose concentrations.

### Distribution of the gap junction protein, Cx43, in ependymal cells is altered under hyperglycemic conditions

**Fig 9:**
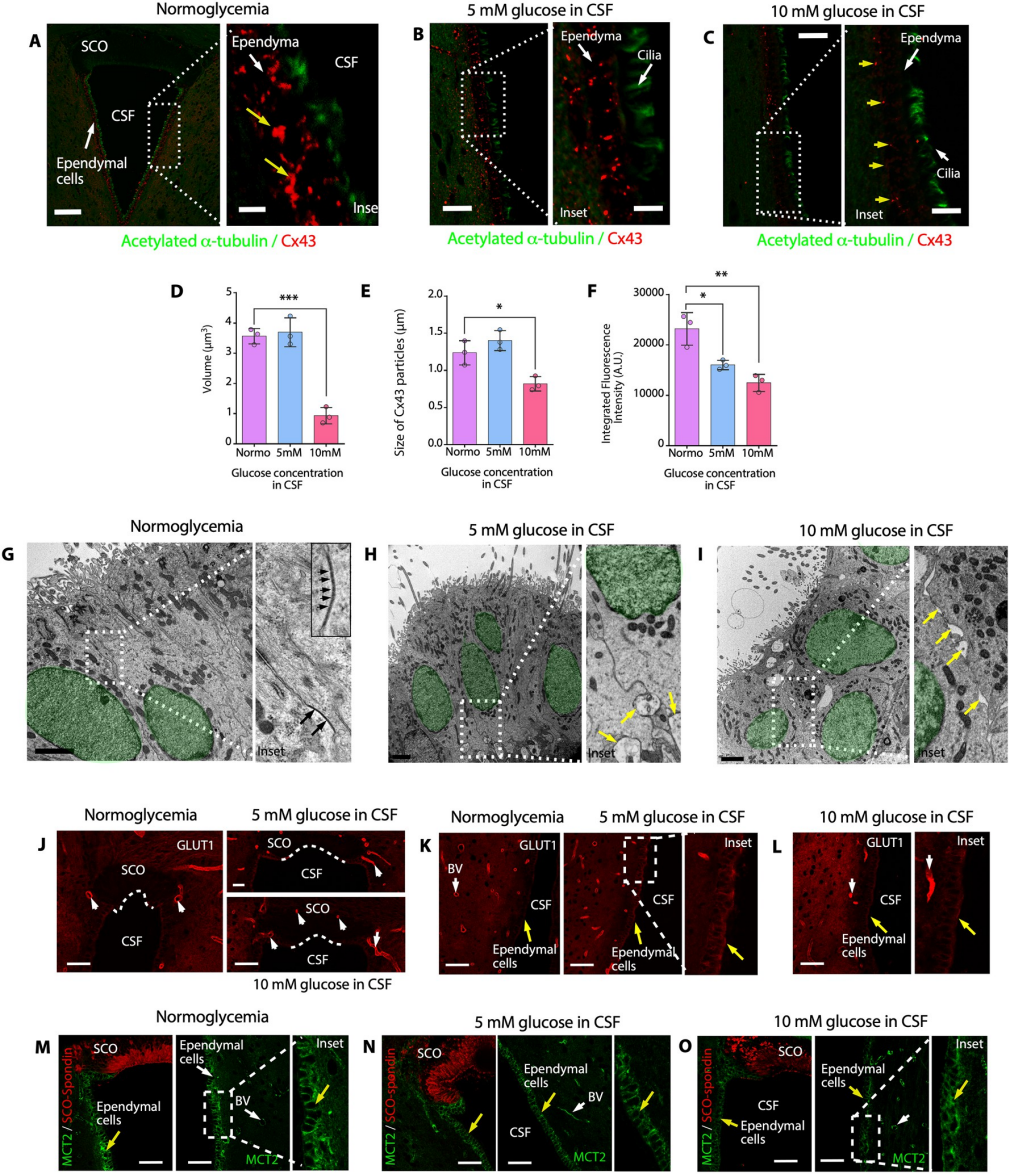
Cx43 is uncoupled from ependymal cells under hyperglycemic conditions; however, GLUT1 and MCT2 do not change the distribution. (**A**) Immunohistochemical staining of acetylated α-tubulin (green) and Cx43 (red) in frontal brain sections from normoglycemic animals. Staining of the dorsal area of the third ventricle was performed. Scale bar: 20 μm. (**B**) Immunohistochemical staining of acetylated α-tubulin (green) and Cx43 (red) in frontal brain sections from 5 mM glucose in CSF hyperglycemic animals. Scale bar: 20 μm. (**C**) Immunohistochemical staining of acetylated α-tubulin (green) and Cx43 (red) in frontal brain sections from 10 mM glucose in CSF hyperglycemic animals. Scale bar: 20 μm. (**D**) Volume of positive Cx43 particles. The graph shows data from 3 biologically independent samples. The error bars represent the SD; ***P < 0.001 (one-way ANOVA). (**E**) Size of Cx43 particles. The graph shows data from 3 biologically independent samples. The error bars represent the SD; *P < 0.05 (one-way ANOVA). (**F**) Integrated fluorescence intensity of Cx43-positive particles. The graph shows data from 3 biologically independent samples. The error bars represent the SD; *P < 0.05, **P < 0.01 (one-way ANOVA). (**G-I**) TEM of ependymal cells from the brains of normoglycemic or hyperglycemic animals. Spaces were observed between the lateral membranes of ependymal cells under hyperglycemic conditions (yellow arrows). Scale bar: 2 μm. The nucleus of each cell is marked in green. (**J-L**) Immunohistochemical staining of GLUT1 (red) in frontal brain sections containing SCO cells and ependymal cells (yellow arrows) from normoglycemic and hyperglycemic rats. No changes in distribution were detected under different glycemic conditions. The blood vessels were highly positive (white arrows). *N* = 3. Scale bar: 30 μm. (**M-O**) Immunohistochemical staining of MCT2 (green) and SCO-spondin (red) in frontal brain sections containing SCO and ependymal cells (yellow arrows) from normoglycemic and hyperglycemic rats. No changes in distribution were detected under different glycemic conditions. SCO cells did not express MCT2. Ependymal cells and blood vessels (white arrows) were highly positive. *N* = 3. Scale bar: 20 μm. Data used to generate graphs can be found in S1 Data. BV, blood vessel; CSF, cerebrospinal fluid; Cx43, connexin-43; d3v, dorsal third ventricle; SCO, subcommissural organ; TEM, transmission electron microscopy.

Recent findings indicate that the Wnt-PLC-IP_3_-Connexin-Ca^2+^ axis maintains ependymal cilia motility in the zebrafish spinal cord; therefore, gap junctions mediate intercellular Ca^2+^ wave propagation, which plays an important role in the maintenance of ependymal cilia motility, and enhancement of gap junction function by pharmacological or genetic manipulation may ameliorate motile ciliopathy [36]. Considering these findings, we hypothesized that under hyperglycemic conditions, the activity of lateral gap junctions in ependymal cells is inhibited via internalization of Cx43, the main connexin expressed in ependymal cells. Under normoglycemic conditions, low-to-absent Cx43 immunoreactivity was observed in SCO cells (Fig 9A); however, in ependymal cells, strong Cx43 immunoreactivity was observed, mainly basolateraly distributed (Fig 9A, insets and yellow arrows). Using 3D superresolution lightning confocal analysis (100 nm optic resolution), we determined the volume, size, and integrated fluorescence intensity of Cx43 particles in ependymal cells under normoglycemic and hyperglycemic conditions (Fig 9D–9F). When the CSF glucose concentration was 5 mM, we observed a change in fluorescent intensity but not in the volume or size of Cx43 particles (*N* = 3) (Fig 9B and 9D–9F). However, when the CSF glucose concentration was 10 mM, the localization of Cx43 in ependymal cells was altered, with immunoreactivity being predominantly localized to the basal area of the cells (Fig 9C, yellow arrows in inset) and changes in the volume, size of Cx43 particles, and fluorescent intensity (Fig 9D–9F) (*N* = 3). These results suggested that by increasing the glucose concentration in the CSF, ependymal cells become laterally uncoupled. Interestingly, TEM revealed the presence of intercellular spaces between ependymal cells in animals under hyperglycemic conditions (Fig 9H and 9I, yellow arrows in insets) (*N* = 3); however, in normoglycemia, the ependymal cells showed nondilated intercellular spaces, with gap junctions between their cells (Fig 9G, inset).

Considering the observed changes in Cx43 levels, we analyzed the levels of transmembrane transporters that are fundamental for the physiological functions of ependymal cells, such as GLUT1 and monocarboxylate transporter 2 (MCT2), to determine whether the intracellular distribution of other membrane proteins is also altered under hyperglycemic conditions (Fig 9J–9L and 9M–9O). However, we did not find any changes in the distribution or expression levels of these transporters. GLUT1 was mainly expressed in the apical regions of ependymal cells (yellow arrows) and capillary endothelia (white arrows) (Fig 9J–9L, insets), and MCT2 was mainly expressed in the basolateral membranes of ependymal cells and endothelial cells of blood capillaries (Fig 9M–9O, insets). These results showed that only the distribution of gap junctions in the lateral membrane of ependymal cells is altered under hyperglycemic conditions.

### SCO-spondin–like protein/WNT5a/Frizzled-2 signaling in human ependymal cells

**Fig 10:**
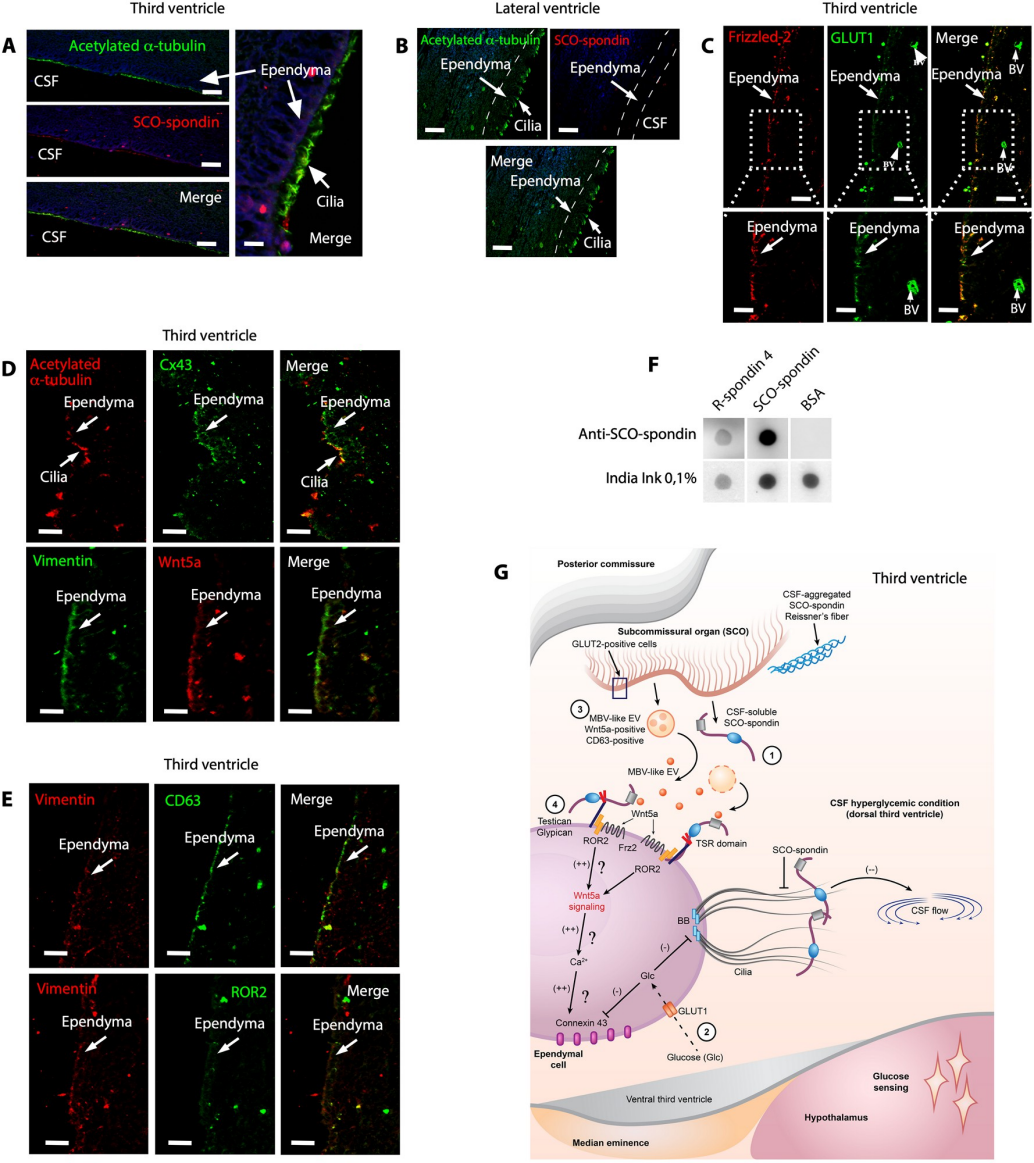
Human ependymal cells express Wnt5a, Frizzled-2, GLUT1, and Cx43 with SCO-spondin–like protein detected in the cilia. (**A** and **B**) Immunohistochemical staining of SCO-spondin (red) and acetylated α-tubulin (green) in ependymal cells from the adult human brain. Scale bar: 15 μm. (**C**) Immunohistochemical staining of Frizzled-2 and GLUT1 in human ependymal cells. Scale bar: 20 μm. (**D**) Immunohistochemical staining of acetylated α-tubulin/Cx43 and vimentin/Wnt5a in human ependymal cells. Scale bar: 20 μm. (**E**) Immunohistochemical staining of vimentin/CD63 and vimentin/ROR2 in human ependymal cells. Scale bar: 20 μm. (**F**) Dot-blot analysis with anti-SCO-spondin. The samples were SCO-spondin and R-spondin-4. (**G**) Overview of hyperglycemic conditions in the third ventricle, SCO cell activation, and the effect of SCO-spondin and Wnt5a on ependymal cells. Similar to β-pancreatic cells, SCO cells express GLUT2, a low-affinity glucose transporter. Under hyperglycemic conditions, the increase in the intracellular glucose concentration is expected to raise the ATP content, stimulating the secretion of SCO-spondin into the CSF (number 1). In ependymal cells, the increase in the glucose concentration changes ciliary beating (number 2). Additionally, it may reduce Cx43-mediated functional coupling. SCO-spondin (soluble in CSF) preferentially binds to the apex of dorsal ependymal cells, further preventing normal ciliary beating. CSF flow shows a transient decline, promoting glucose sensing in the basal hypothalamus (bottom pink area). When the glucose level in the CSF is high, SCO cells also release Wnt5a, possibly via CD63-positive MVB-like EVs (number 3). Wnt5a bound to ROR2 (very likely anchored to testican or glypican) can be internalized (number 4) [38,40] and probably activating the noncanonical β-catenin signaling pathway (number 4) [36]. CX43 is uncoupled under hyperglycemic conditions, and this alteration may be reversed when the intracellular calcium increases [36]. The original blot for this figure (F) can be found in S1 Raw Images. BV, blood vessel; CSF, cerebrospinal fluid; Cx43, connexin-43; EV, extracellular vesicle; MVB, multivesicular body; ROR2, Frizzled 2/receptor tyrosine kinase-like orphan receptor-2; SCO, subcommissural organ.

Because the SCO is not present in the adult human brain, we speculate that ependymal cells may acquire the function of this brain region. We suppose that this mechanism may be essential for the maintenance of CSF glucose homeostasis, preventing pathological alterations related to hyperglycemia in the ventricles, such as ventricular edema. We analyzed adult human brain tissue samples and found that acetylated α-tubulin was expressed in ependymal cells of the dorsal third ventricle, specifically in ependymal cell cilia. Furthermore, we found weak SCO-spondin–like protein immunoreaction, mainly in cilia (Fig 10A). Only acetylated α-tubulin was observed in ependymal cells in the lateral ventricles (Fig 10B). Additionally, we detected GLUT1 in the apical region of ependymal cells and endothelial cells (Fig 10C, inset). Moreover, GLUT1 was colocalized with Frizzled-2 in ependymal cells but not the capillary endothelium (Fig 10C, inset). Additionally, ependymal cells were positive for Cx43 and Wnt5a, and these proteins were mainly expressed intracellularly (Fig 10D). Finally, we observed CD63 immunoreactivity (Fig 10E) and weak ROR2 immunoreactivity (Fig 10E). We concluded that most of the proteins previously identified in rat ependymal cells (synthesized by them or bound to them) could be detected in human third ventricle ependymal cells.

To further identify the proteins expressed in human ependymal cells, we analyzed the expression of different genes using a database of human ciliated ependimoma cells (CECs) samples and through alignment projection of scRNA-seq data from 26 pediatric patients [41] (S10A–S10O Fig). From the database, we found that Wnt5a, Frizzled-3, Frizzled-5, Frizzled-6, ROR2, testican-2, and glypican-1 were expressed in CEC cells (S10B–S10H Fig); GLUT1, MCT1, MCT2, and Cx43 were also expressed in these cells (S10I–S10L Fig). Surprisingly, the mRNA for SCO-spondin was detected in a few CECs; however, spondin 2 and R-spondin 4 were more frequently detected (S10M–S10O Fig). R-spondin 4 was detected in 34% of all cells analyzed [41]. Thus, we tested whether anti-SCO-spondin reacts positively with purified human R-spondin-4. Due to the high number of TSR domains in bovine SCO-spondin, it is likely that the antiserum can recognize the spondin cystine-rich domains. Achieving dot-blot analysis (Fig 10F), we confirmed that anti-SCO-spondin can recognize human R-spondin-4.

Here, we demonstrated that the secretion of SCO-spondin by SCO cells is increased under hyperglycemic conditions. Additionally, SCO cells secrete CD63-positive vesicles and Wnt5a, which are attached to ependymal cell cilia. SCO-spondin regulates ciliary beating under hyperglycemic conditions, and Wnt5a may activate ciliary movement under normoglycemic conditions by stimulating cell coupling mediated by Cx43 [41]. Most of the proteins detected in the rat brain were also detected in third ventricle ependymal cells of the human brain. Finally, we propose an overview of hyperglycemic conditions in the third ventricle, SCO cell activation, and the effect of SCO-spondin and Wnt5a on ependymal cells (Fig 10G).

## Discussion

Two decades ago, we reported that GLUT2 is expressed in hypothalamic tanycytes, demonstrating that these cells participate in the brain’s glucose-sensing system, metabolizing glucose and generating lactate, which is delivered to neurons in the arcuate nucleus [4,42]. This concept was recently confirmed with genetic tools and transgenic animals [2]. Additionally, we have shown that glucose is directly transferred from fenestrated capillaries in the median eminence (ME), which lacks a blood–brain barrier structure, to the basal hypothalamus CSF, increasing the local glucose concentration [1]. Furthermore, it has been widely reported that choroid plexus cells transport glucose to the dorsal region of the ventricular system, impacting neighboring structures such as the SCO [43,44]. The glucose that reaches the basal third ventricle is captured by tanycytes for its metabolization and glucose sensing mechanism activation. Undoubtedly, temporarily generating a reduced CSF flow would enhance this glucose-sensing mechanism, which begins with the transfer of glucose to the CSF at ME and subsequently impacts hypothalamic tanycytes and neurons [1].

Here, we expand the current knowledge on the glucose-sensing mechanism in the brain, showing that the SCO, another circumventricular organ with unknown function in the adult brain, is involved in this mechanism [9,12]. As GLUT2 is expressed in SCO cells (Fig 1), we postulate that the SCO acts as a glucose sensor. Thus, this hypothesis was tested by exploring the effect of increasing glucose concentration in the CSF from 3 mM (control) to 5 or 10 mM (hyperglycorrhachia). Our data indicate substantial changes in SCO cell secretion, resulting in accelerated release of SCO-spondin, probably through a “pulse” that causes the release of apical secretory granules contents from epithelial cells (Fig 10G, number 1) and, additionally, increases the solubilization of previously secreted and extracellularly semipolymerized SCO-spondin. The presence of low-affinity glucose transporters, such as GLUT2, would allow continuous glucose uptake, which could eventually lead to rapid increases in ATP concentration, as has been previously described in tanycytes and β-pancreatic cells [2,4,25]. Additionally supporting this hypothesis, previously it was found that an increase in intracellular ATP content in SCO cells elicited rises in [Ca^2+]^i in 85% of analyzed cells [24]. These effects were dose dependent and involved NK_3_ and P_2_Y_2_ receptors linked to G protein and PLC activation. In all ATP-sensitive cells, the increase in the [Ca^2+^]i involves calcium release from thapsigargin-sensitive intracellular stores and PKC-mediated influx of extracellular calcium via L-type voltage-gated calcium channels (VGCCs) [24]. Thus, SCO cells would have the capacity to respond to increased glucose concentrations, increasing their apical secretion and SCO-spondin solubilization in CSF.

Under normoglycemic conditions, SCO cells release SCO-spondin into the CSF via exocytosis. We postulate that constitutive secretion results in the aggregation and polymerization of products that form RFs, because SCO-spondin was not observed on the surface of ependymal cells. Conversely, when the CSF glucose concentration is increased, differential SCO-spondin secretion from SCO cell blebs was observed, and secreted SCO-spondin was detected on ependymal cell cilia (Figs 4D–4F and 5F and 5G). According to the aforementioned data, we postulate the existence of an “integrated circumventricular system for glucose sensing” under hyperglycemic conditions. Thus, even small changes in the glucose concentration induced by the choroid plexus cells and ME in the CSF induce the release of SCO-spondin, which binds to the cilia of dorsal ependymal cells. Hyperglycemia in CSF increases the glucose concentration in ependymal cells, decreasing ciliary beating, which is enhanced by the interaction of SCO-spondin with dorsal ventricular cilia (Figs 6E and 10G, number 2). Furthermore, at higher CSF glucose concentrations, we postulate that the coupling of ependymal cells via gap junctions (Cx43) may be reduced; however, further functional experiments are needed to demonstrate this observation. Gap junctions play an important role in the coordination of Ca^2+^ oscillations in adjacent ependymal cells, and gap junctions are required for coordinated beating [36]. Thus, a higher CSF glucose concentration allows CSF to move more slowly in the dorsal third ventricle, which is very likely to be required for maintaining CSF at the basal hypothalamus for a longer time, increasing glucose-sensing activity.

Surprisingly, we observed that SCO cells secrete CD63-positive exosome-type vesicles, which may eventually remain attached to SCO-spondin–forming RFs [9] or interact with unpolymerized SCO-spondin, which combines with ependymal cells. Consequently, vesicles release Wnt5a to maintain the local concentration of this protein in ependymal cells, where it would have a chance to interact with HSPGs, glypican, and/or testican (Figs 7 and S9). Lipid modification of Wnt proteins [45] represents a challenge for the transport of Wnts in water-based extracellular fluids, such as CSF. Different mechanisms for long-range transport of Wnts, such as transport via lipoprotein particles [46], incorporation into exosomes [47–49], and direct binding to the transporter protein Swim [50], have been proposed in *Drosophila*. Wnts have been found to be transported via exosomes in the epididymal fluid of mice [51] and by binding to the transport protein, Afamin, in vitro [52,53]. Additionally, it was recently reported that Wnt5a is secreted into the CSF by hindbrain embryonic choroid plexus cells, where it is preferentially associated with lipoprotein particles rather than exosomes, allowing it to exert biological effects from a distance [37]. Similar mechanisms could be present in the adult brain.

We postulate that Wnt5a secreted by SCO cells is release via CD-63–positive exosome-like vesicles, which interact with SCO-spondin in the first stage (Fig 10G, numbers 3 and 4). Once close to ependymal cells, Wnt5a is released and interacts with ROR2/Frizzled-2, both of which are expressed in ependymal cells, and the interaction between Wnt5a and ROR2 or Frizzled-2 is expected to activate noncanonical β-catenin signaling (Fig 10G, number 4). Because ependymal cells are partially uncoupled under hyperglycemic conditions, Wnt5a signaling may induces the formation of gap junctions by Cx43, which couple ependymal cells to activate ciliary beating [54] when glucose levels begin to be normalized. However, the action of Wnt5a on the ciliary beating of ependymal cells of the third ventricle should be analyzed with new experiments. A similar pathway associated with the ciliary movement of ependymal cells in the central canal of the spinal cord was recently proposed [36]; the researchers proposed that the Wnt-PLC-IP_3_-Connexin-Ca^2+^ axis maintains ependymal cilia motility in the zebrafish spinal cord. The abovementioned mechanism occurs in the dorsal region of the third ventricle and acts as a secondary regulatory of CSF flow, similar to the effect of MCH on ciliary beating in the ventral ventricle [8]. In recent years, studies have shown that ciliary beating can be modulated by different physiological processes; therefore, increased CSF glucose concentration and SCO-spondin secreted by SCO cells may coordinate CSF flow to regulate glucose sensing and feeding behavior under hyperglycemic conditions in the basal third ventricle.

We propose a new mechanism underlying the regulation of ciliary beating in ependymal cells that allows functional coupling of 3 circumventricular organs (the SCO, choroid plexus, and hypothalamic ME) to increase glucose sensing in the hypothalamus. One of the limitations of our study is that we did not further examine whether SCO cells and SCO-spondin play a glucose-sensing role under other circumstances. One future direction is to investigate how these proteins modulate ciliary beating in diabetes, obesity, or in alcohol consumption [5–7], all conditions that can alter ependymal cell function.

At present, it is unknown whether this mechanism is present in the adult human brain, which has no SCO cells, thus discussing a replacement mechanism can be highly speculative. However, we suggest in this study that human ependymal cells may express SCO-spondin-like protein(s). The ependymal cell could secrete R-spondin-1-4, proteins that have cysteine-rich/furin-like domain, thrombospondin domain, and a C-terminal basic region [55]. It has been postulated that this protein may regulate the canonical Wnt/beta-catenin–dependent pathway and noncanonical Wnt signaling by acting as an inhibitor of ZNRF3, an important regulator of the Wnt signaling pathway [55]. R-spondins 1–4 can directly bind to RNF43/ZNRF3 and facilitate their removal from the cell membrane, thus increasing membranous frizzled receptors and sensitizing cells to respond to WNT [56]. Of all the R-spondins studied, R-spondin-4 is highly expressed in the brain and lung (Human Protein Atlas; [57]). It has also been detected in human ependymoma cells [41] and in different types of glial cells (Human Protein Atlas; [57]). In our study, R-sponding-4 was recognized by the anti-SCO-spondin antibody, eventually through the cysteine-rich domain. Therefore, R-sponding-4 should be considered as a fundamental protein in the eventual mechanism proposed for the human brain at the ependymal cell. Furthermore, we propose that human ependymal cells can secrete Wnt5a (or a similar protein) to regulate ciliary beating in ependymal cells. Wnt5a, Frizzled-2, Cx43, and testican/glypican, which are involved in this mechanism, are also expressed in the human brain ependymal cells. Wnt5a has been widely detected ubiquitously in the human brain, and Frizzled-2 has been highly expressed in human choroid plexus ependymal (Human Protein Atlas; [57]). Alignment projection of scRNA-seq data from 26 pediatric patients with EPN showed that all these molecules are expressed in human ciliary ependymomas (EPNs) (Figs 10 and S10) [41]. Due to the absence of the SCO in the adult human brain, we suggest that under hyperglycemic conditions, ciliary beating is regulated for an autocrine manner in ependymal cells and by glucose secreted by the hypothalamic ME and choroid plexus cells. This mechanism may allow a comprehensive understanding of the physiological and molecular mechanisms related to glucose sensor systems in the brain and the regulation of feeding behavior and provides an understanding of associated pathologies, such as obesity and diabetes, and their impact on the physiology of different circumventricular organs and ependymal cells.

## Methods

### Ethics statement

All experiments and facilities were approved by the Committee for Ethics of Animal Experiments and were conducted in conformity to the Guidelines for Animal Experiments, Concepción University (number 11150678)—Manual of Biosafety Standards and Associated Risks, CONICYT 2009 and Málaga University (CEUMA 37-2015-A); Consejería Agricultura, Pesca y Alimentación Ref 2016/11402. The procedures in Swiss Webster mice were approved by the veterinary office of the Canton de Vaud (Switzerland).

### Animals

Two-month-old male Sprague–Dawley rats were used in most experiments, and 1.5-year-old rats were used in the remaining experiments. The average weight of the adult rats was 275 ± 25 g. The animals were killed at the beginning of the light period (9:00 to 12:00). Additionally, 16-week-old Swiss Webster mice and 8-week-old *Slc2a2*^*loxP/loxP*^ mice were generated as previously described [58]. For all experiments, only males were used. Both strains were given ad libitum address to rodent chow (Lab Diet, Animal Care, St. Louis, MO, USA) except during the experiments. A total of 4 to 5 mice were housed per cage at temperature 25°C on a 12-h/12-h light/dark cycle.

### Glucose administration and glycorrhachia determination

Two-month-old rats were fasted for 48 h before glucose injection. Low hyperglycemia was induced by IP injection of 0.5 g/kg body weight glucose to achieve a glucose level of 17 ± 1.7 mM after 30 min (*N* = 4). High hyperglycemia was induced by IP injection of 2 g/kg body weight glucose to achieve a glucose level of 29.5 ± 6.2 mM (*N* = 11) after 30 min. The control animals showed normal glucose level, i.e., 7.25 ± 2.1 mM (*N* = 6). The CSF glucose concentration for each condition is presented in the results section. For stereotactic methods for glycorrhachia determination, the animals were anesthetized, and a cannula guide (PlasticsOne, San Diego, CA, USA) was implanted using the following brain stereotaxic parameters: third ventricle, − 3.14 mm, lateral 0.0 mm, and dorsal-ventral 9.2 mm. After 3 days of recovery, we induced normoglycemia and hyperglycemia, and glycorrhachia was determined in CSF isolated from the third ventricle. A sample of 2 μL was collected for this procedure, and the glucose determinations were performed using Accucheck test strips (Roche Applied Science, Basel, Switzerland). For CSF glucose determination, a factor correction of 1.06 from a calibration curve using artificial CSF was applied.

Additionally, 3 rats with normal glucose concentrations were anesthetized with 5% isoflurane (Forane, Baxter Corporation, Ontario, Canada) and injected intravascularly with 225 μL of 50 mM 2-NBDG (Thermo Fisher Scientific, Waltham, MA, USA) [59]. After 15 min, the animals were transcardially perfused with paraformaldehyde, and the brains were obtained and sectioned using a vibratome for analysis of ventral third ventricle ependymal cells and SCO cells.

*Slc2a2*^*loxP/loxP*^ mice were administered 30% glucose by IP injection to induce hyperglycemia (2 weeks after virus transduction; see below). After 40 min, the mice were anesthetized with 5% isoflurane (Forane, Baxter Corporation) and perfused with 2.5% glutaraldehyde and 2% paraformaldehyde for TEM or 4% paraformaldehyde for immunofluorescence. The average glucose concentration of the control animals was 7.49 ± 0.7 mM (*N* = 3). The average glucose level in mice with hyperglycemia was 18.3 ± 3 mM (*N* = 6).

### Viral vector delivery

Eight-week-old *Slc2a2*^*loxP/loxP*^ mice were anesthetized with IP injection of a ketamine/xylazine (80 mg/kg/12 mg/kg) cocktail. Loss of reflexes was assessed by pinching the hind limb. Subsequently, the mice were positioned on a stereotaxic instrument (RWD Life Science). A total of 0.2 μL of AAV_5_-GFAP-GFP (9.1 × 10^12^ particles per mL) (UNC Vector Core, North Carolina, USA) or AAV_5_-GFAP-Cre-GFP (4.9 × 10^12^ particles per mL) (UNC Vector Core) was injected into the brain at the following stereotaxic coordinates: −2.54 mm AP, 0.0 mm ML, and −2.5 mm DV. The virus was injected using a microinjection syringe pump (Word Precision Instruments, Sarasota, FL, USA) at a flow rate of 0.1 μL min^−1^. Subsequent experiments were performed 2 weeks after virus transduction.

### Immunohistochemistry, immunofluorescence, and confocal microscopy

Adult rats or 8-week-old virus-injected *Slc2a2*^*loxP/loxP*^ mice were anesthetized with 5% isoflurane (Forane, Baxter). Subsequently, the animals were transcardially perfused with phosphate buffer (pH 7.4; PBS, 137 mM NaCl, 2.7 mM KCl, 8 mM Na_2_HPO_4_, and 1.5 mM KH_2_PO_4_) followed by 10 (mice) or 30 (rat) mL of paraformaldehyde 4% or Bouin solution. The tissues were stored in fixative for 24 h after perfusion. The rat brains were embedded in paraffin blocks, and the tissues were washed with distilled water, dehydrated in graded ethanol solutions (70%, 95%, and 4 times in 100%), and cleared. Subsequently, the samples were embedded in paraffin. Finally, the paraffin blocks were cut with a rotatory microtome (Reichert-Jung 2040) into 7-μm sections and mounted on Superfrost TM Plus slides (Thermo Fisher). The brains of *Slc2a2*^*loxP/loxP*^ mice were embedded in 30% sucrose solution for 72 h.

Then, the samples were embedded in Neg-50 cryopreservative solution (Thermo Fisher Scientific) and stored at −80°C until processing. Using a cryostat (Microm HM520, Thermo Fisher Scientific), 40-μm frontal brain sections were obtained. For immunohistochemistry and DIC imaging, rat frontal brain slices (7 μm) were incubated with hydrogen peroxide at 3% v/v in methanol for 30 min to inactivate endogenous peroxidase and treated using standard methods [60]. For immunofluorescence, mouse brain tissue sections (obtained by a freezing microtome) were used for multiple labeling for confocal microscopy and DIC. The sections were washed 3 times with 10 mM Tris-phosphate buffer (10 mM Tris, 120 mM NaCl, 8.4 mM Na_2_HPO_4_, and 3.5 mM KH_2_PO_4_ (pH 7.8)) and incubated with primary antibody (see above) prepared in 10 mM Tris-phosphate buffer (pH 7.8) supplemented with 1% w/v bovine serum albumin (BSA) in a humid chamber for 16 h. After 3 washes for 10 min each, the sections were incubated for 2 h at room temperature (RT) with goat anti-rabbit immunoglobulin (IgG) coupled to Alexa Fluor 488 (Jackson ImmunoResearch Laboratory, Baltimore Pike, PA, USA), goat anti-chicken IgY (H+L) coupled to Alexa Fluor 647 (Jackson ImmunoResearch Laboratory), or goat anti-mouse IgG (H+L) coupled to cyanine Cy3 (Jackson ImmunoResearch Laboratory). All secondary antibodies were prepared in 10 mM Tris-phosphate buffer supplemented with BSA. Additionally, Hoechst 33342 was used for nuclear staining. As a negative control, the primary antibody was omitted. The tissue samples were analyzed with an LSM 780 NLO spectral confocal microscope (Zeiss). Images were acquired with Zen 2011 software (Zeiss, Berlin, Germany) after Z-stack and tile-scanning analysis. Imaris software was used for rendering. For optical microscopy following immunoperoxidase or immunofluorescence analysis, at least 15 7-μm sections of each area of the SCO were analyzed. Tissues were sectioned with a freezing microtome (40 μm) for confocal microscopy, and 6 to 8 sections per animal covering a thickness of approximately 400 μm were analyzed. Samples of postmortem human brain tissue from healthy controls were obtained from the Harvard Brain Tissue Bank and fixed directly by immersion in 4% (w/v) paraformaldehyde. The use of unidentified brain tissue samples was exempted under the regulations of the Office for the Protection of Research Subjects (University of Illinois Chicago).

### Reagents and antibodies

The following primary antibodies were used in this study: rabbit anti-bovine RF compounds (SCO-spondin) (1:1,000 dilution; produced in-house at Málaga University), chicken anti-human vimentin (AB5733, 1:400 dilution, Millipore, Billerica, MA, USA), rabbit anti-human GFAP (MAB360, 1:500 dilution, Millipore), mouse anti-βIII tubulin (G712A; 1:1,000 dilution; Promega, Madison, Wisconsin, USA), rat anti-mouse frizzled-2 (sc-74019, 1:100 dilution; Santa Cruz Biotechnology, Santa Cruz, CA, USA), mouse anti-human Wnt5a (sc-365370, 1:100 dilution; Santa Cruz Biotechnology), rat anti-mouse Wnt5a (MAB645, 1:200, R&D System, Minneapolis, MN, USA), rabbit anti-human GLUT1 (C110491, 1:100 dilution, EMD Millipore, Burlington, MA, USA), rabbit anti-rat GLUT2 (GT21-A, 1:100 dilution; Alpha Diagnostic, San Antonio, TX, USA), rabbit anti-human GLUT6 (GT62-A, 1:100 dilution; Alpha Diagnostic), mouse anti-human β-catenin (sc-7963, 1:200 dilution; Santa Cruz Biotechnology), mouse anti-human ROR2 (sc-374174, 1:200 dilution; Santa Cruz Biotechnology), mouse anti-human CD63 (sc-5275, 1:200 dilution; Santa Cruz Biotechnology), mouse anti-human aggrecan (4F4) (sc-33695, 1:200 dilution; Santa Cruz Biotechnology), mouse anti-human Syndecan-2 (sc-365624, 1:200 dilution; Santa Cruz Biotechnology), mouse anti-human HS6ST1 (sc-398231, 1:200 dilution; Santa Cruz Biotechnology), mouse anti-human testican 2 (sc-515691, 1:200 dilution; Santa Cruz Biotechnology), mouse anti-human glypican-1 (sc-365000, 1:200 dilution; Santa Cruz Biotechnology), mouse anti-human MCT2 (sc-166925, 1:200 dilution; Santa Cruz Biotechnology), rabbit anti-Cx43 (C6219, 1:300 dilution: Millipore, Billerica, MA, USA), mouse anti-KDEL (sc-58774, 1:200 dilution; Santa Cruz Biotechnology). Additionally, we used CellMask (0.3X ex/em (nm) 650/655) (Invitrogen, Waltham, MA, USA) deep red plasma membrane stain for in vivo analysis.

### CLARITY protocol

This technique was performed according to a previously described CLARITY protocol with modifications [61]. The rats were transcardially perfused with 20 mL of cold hydrogel composed of 4% v/v acrylamide, 0.005% v/v bis-acrylamide, 0.025% p/v initiator VA-044, 10% v/v PBS, and 4% p/v paraformaldehyde. The brains were incubated in a 50-mL tube with 20 mL of hydrogel and refrigerated at 4°C for 2 days. Subsequently, the tissues were desiccated in an extraction chamber filled with nitrogen with a vacuum pump. A vacuum was maintained for 10 min, and the nitrogen was allowed to enter the tube with the sample. The desiccator was opened, and the tube containing the sample was hermetically closed, minimizing exposure to oxygen (since it prevents the polymerization of the hydrogel). The samples were incubated at 37°C with rotation for 3 h. Then, 1.5-mm thick sagittal sections were cut with a vibratome and incubated with 10 mL of clearing solution (0.2 M boric acid and 40% w/v sodium dodecyl sulfate (pH. 8.5)) 7 to 10 days at 37°C with stirring at 225 rpm. Subsequently, the rinsed slices were washed in PBST (PBS 1X with 0.1% v/v Triton-X 100) for 2 days. After the slices were rinsed, immunostaining was performed. The tissues were incubated with primary antibody diluted 1:500 for 2 days and washed with PBST several times for 1 day after which they were then incubated with secondary antibody diluted 1:200 for 2 days and washed once with PBST for 1 day. Subsequently, the samples were placed in 80% v/v glycerol for at least 3 h before being visualized under a microscope. After mounting, the confocal and two-photon imaging system (Zeiss) was used for imaging. The images were analyzed with Zen software (Zeiss AIM Software, Berlin, Germany).

### In vivo brain magnetic resonance imaging (MRI)

The brains of 3.5-month-old hyperglycemic and control rats were imaged by MRI under anesthesia [ketamine (91 mg/kg) and xylazine (9.1 mg/kg), IP] using a BioSpec 9.4T Bruker animal MRI system (Bruker, Manning Road Billerica, MA) with a transmitting and receiving head resonator of 2.5 mm. The rats were anesthetized and placed in a cradle equipped with a pressure probe to monitor the respiratory rate. The body temperature was maintained at 37°C using magnet gradients. T2-weighted images were acquired in the axial plane using a spin-echo sequence (echo time = 37.1 ms, repetition time = 5,000 ms, rapid acquisition with a relaxation enhancement factor of 8,4 averages).

### Transmission and scanning electron microscopy

Sections (90 or 150 μm) fixed in 4% paraformaldehyde and 2.5% glutaraldehyde were rinsed in 0.1 M phosphate buffer [62] and then postfixed in 2% osmium tetroxide for 1 h. After the sections were rinsed, they were stained with 2% uranyl acetate in 70% ethanol for 3 h, dehydrated in ascending concentrations of alcohol, and incubated with propylene oxide for Araldite embedding. Once plasticized, the sections were cured at 60°C for 3 days. Serial semithin sections (1.5 μm) were cut on an ultramicrotome (Leica, Wetzlar, Germany) and then stained with 1% toluidine blue. Subsequently, ultrathin (60 nm) sections were cut with a diamond knife using the same ultramicrotome and examined under a Jeol Jem-1400 electron microscope (Jeol, Peabody, MA, USA). For scanning electron microscopy, the animals were fixed in 4% paraformaldehyde and 2.0% glutaraldehyde in 0.1 M phosphate buffer [62] and then postfixed in 1% osmium tetroxide for 1 h. After standard procedure, the samples were examined under a Tescan Vega scanning electron microscope (Tescan, Brno—Kohoutovice, Czech Republic).

### Gold immunostaining after intraventricular antibody perfusion

Two-month-old male Sprague–Dawley rats were used in the present experiment. All animals were anesthetized with 5% isoflurane (Forane, Baxter). Subsequently, via a cannula stereotaxically implanted into the right lateral ventricle, 5 μl of anti-SCO-spondin were perfused at a rate of 1 μl /min, by using a perfusion pump. The control rats were perfused with rabbit-IgG (*N* = 3). Coordinates for the cannula placement within the lateral ventricle were posterior from Bregma = 0.5 mm; lateral from sagittal suture = 1.8 mm; ventral from dura = 4.0 mm. The rats were maintained with intraventricular antibody for 30 min. Once the perfusion was started, the rats were injected IP with glucose to achieve glycorrhachia concentrations of 3 mM (*N* = 3, controls), 5 mM (*N* = 3), or 10 mM (*N* = 3). An additional control rat only perfused with rabbit-IgG was also performed.

For immunogold electron microscopy, preembedding immunogold staining was performed as previously described [62]. Briefly, rats were deeply anesthetized with 250 mg/kg body weight tribromoethanol (Avertin, Chemos GmbH, Regenstauf, Germany) and perfused transcardially with saline (0.9% NaCl), followed by 2% paraformaldehyde and 0.5% glutaraldehyde in 0.1 M phosphate buffer. Brains were removed, postfixed by immersion in same fixing solution at 4°C overnight, and cut into 150 μm semithin sections using a vibratome. The sections were incubated with secondary donkey anti-rabbit IgG conjugated to colloidal ultrasmall gold particles (#25801, EMS, Hatfield, PA, USA) at a 1:50 dilution for 24 h, at 4°C. After enhancement of gold particles with silver, sections were washed, postfixed with 2.5% glutaraldehyde for 20 min, washed, and finally postfixed with 1% osmium tetroxide for 30 min. During dehydration, sections were stained with uranyl acetate. The tissue sections were embedded in Araldite 502 (EMS). Ultrathin sections (60-nm thickness) were analyzed with an electron microscope (Philips CM100).

### Ependymal flow assay

Sprague–Dawley rats were killed according to bioethical manuals. Whole-mounts containing the dorsal third ventricle were freshly dissected and placed in aCSF (1 mM NaH_2_PO_4_ (pH 7.3), 119 mM NaCl, 26 mM NaHCO_3_, 2.5 mM KCl, and 1.3 mM MgCl_2_) containing 3 mM glucose at 37°C. The 3-mM glucose concentration represents the control in our experiments. The brain sections were attached to glass-bottom dishes for live-cell imaging (WillCo Wells, B.V., Amsterdam, the Netherlands). Fluoresbrite Carboxy YG 2.0 Micron Microspheres (Polyscience, Warrington, PA, USA) were diluted 1:100 in aCSF containing glucose at different concentrations (3 mM, 5 mM, and 10 mM) and deposited onto the ventricular surface using a Hamilton syringe, which reduces flow. Additionally, the cells were also treated with SCO-spondin solubilized from bovine RF [63,64]. Ependymal flow assays were performed with a Leica SP8 confocal microscope equipped with an 8-kHz resonant scanner and hybrid detectors (HyDs) at a controlled temperature of 37°C and atmosphere of 5% CO_2_. Images with a size of 512 × 512 were acquired in unidirectional mode in the x, y, and t dimensions at a speed of 30 fps for 120 s using an HC PL APO CS2 20×/0.75 DRY objective. The speed of the migrating fluorescent beads was quantified using IMARIS v9.2 software (Bitplane, Concord, MA, USA).

### Protein dot blot

Recombinant human R-Spondin 4 (R&D Systems, Minneapolis, MN, USA) was used to evaluate the cross-reactivity of anti-SCO-Spondin antibody through dot blot. Bovine SCO-spondin, BSA, and R-spondin 4 (1 μg) were seeded on activated Inmobilon-P (PVDF) membranes (Merck Millipore) and washed with TBS 1× buffer, 0,05% Tween-20 (TBS-T), and blocked with TBS-T, 5% BSA for 30 min at RT. The primary antibody was incubated overnight in TBS-T, BSA 0,1% at 4°C. Membranes were washed with TBS-T, BSA 0,1% and incubated with HRP-conjugated anti-Rabbit IgG (1:5,000, Jackson ImmunoResearch Laboratory), in TBS-T, BSA 0,1% for 2 h at RT. Membranes were rinsed 3 times with TBS-T and revealed by Lighting Plus ECL reagent (Perkin Elmer, Waltham, MA, USA) using the ImageQuant LAS500 enhanced chemiluminescence system (General Electric, Austin, TX, USA).

### Image processing and quantitative analyses

Images were acquired with the same set of parameters between conditions using LSM780 Zeiss confocal microscope and SP8 Leica lightning confocal microscope. The mean fluorescence intensity (MFI) was quantified using ImageJ software (NIH). Positive staining regions of interest (ROIs; at least from 3 independent samples) in the SCO and ependymal layers were selected for both control and hyperglycemic conditions to measure the MFI for GLUT1, GLUT2, GLUT6, SCO-spondin, KDEL, CD63, acetylated α-tubulin, Wnt5a, Frizzled-2, and Cx43 staining. Additionally, in some samples, the cilia length positive for acetylated α-tubulin was measured manually using the Profile tool from ZEN software (LSM780 Zeiss confocal microscope). The orientation of acetylated α-tubulin immunoreaction in cilia images after CLARITY was analyzed by ImageJ software and directionality plug-in (NIH). The “Amount” column is the sum of the histogram from center−std to center+std, divided by the total sum of the histogram. The real histogram values are used for the summation, not the gaussian fit. For colocalization analysis, IMARIS 9.2 software (Bitplane) was used, and Mander’s overlap coefficient was graphed.

To perform the quantification of Cx43 in Fig 9, the images were first acquired in x, y, z using the Leica SP8 superresolution confocal microscope equipped with the Lightning module. Then, the files containing the 3D sample information were exported in lif format and reconstructed in the Imaris software. Finally, an ROI of the same size was selected for the different experimental conditions and fluorescence was quantified per unit area (integrated intensity). Therefore, the graph indicates integrated fluorescence intensity (AU) on the y-axis.

### Statistical analysis

The data are presented as the means ± standard deviation (SD). In our study, N corresponds to biological replicates. Comparisons between two groups were made using one-tailed unpaired Student *t* test. For comparison of more than 2 groups, standard one-way ANOVA with a post hoc Tukey multiple comparison test was applied. A *p* ≥ 0.05 value was considered statistically significant. It is important to highlight that no experimental subjects were excluded from the statistical analysis. All statistical analyses were performed using Prism 6.0 software (GraphPad Software, San Diego, CA, USA).

## Supporting information

S1 Fig SCO cells do not show a constricted morphology under normoglycemic conditions. (**A, B**) Tile-scanning TEM. Under normoglycemic conditions, SCO cells did not exhibit structural changes, were normally polarized, and had dilated ER cisternae (A, ER area), and apical areas with blebs containing secretory granules, microvilli and cilia (B, C, D, black arrows). MVB, most of them containing few vesicles (E), were also detected. Scale bar: A, 10 μm; B, ER and nuclear areas, 2 μm; C to E, 0.2 μm. *N* = 3. BB, basal body; BV, blood vessel; ER, endoplasmic reticulum; MVB, multivesicular body; SCO, subcommissural organ; TEM, transmission electron microscopy.
(TIF)

S2 Fig Hyperglycemia (CSF glucose concentration of 5 mM) increases the number of SCO cells with constricted morphology. (**A-C**) TEM analysis when the CSF glucose concentration was 5 mM. SCO cells with constricted morphology and intercellular spaces in areas close to the ventricle were observed. Scale bar: A, 10 μm; B, 2 μm; C, 4 μm. (**D**) Apical region of SCO cells. We detected blebs, cilia, and microvilli and extracellularly disaggregated SCO-spondin. MVBs-like EV were secreted (D, insets). Exosome-like vesicles intermixed with secreted SCO-Spondin and cilia were also observed (arrows and insets). Scale bar: D, 1 μm; higher magnification, 0.1 μm. (**E**) Dorsal ependymal cells. Scale bar: 5 μm. (**F**) Ependymal cell cilia. Floccular material associated with cilia membranes (arrow and insets). Scale bar: 0.5 μm; higher magnification, 0.2 μm. (**G**) Choroidal plexus cells showed a normal structure without secretory material outside the cells. Scale bar: 3 μm; higher magnification, 1 μm. *N* = 4. CSF, cerebrospinal fluid; EV, extracellular vesicle; MV, microvilli; MVB, multivesicular body; SCO, subcommissural organ; TEM, transmission electron microscopy.
(TIF)

S3 Fig Hyperglycemia (CSF glucose concentration of 10 mM) increases SCO cell constriction and MVB-like EV secretion. (**A**) Tile-scanning TEM. SCO cells were contraction, giving the impression of a “pricked” tissue. Scale bar: A, 5 μm. (**B** and **C**) TEM revealed no apparent structural changes in the posterior commissure (axons or blood vessels) or ependymal cells. No subependymal edema was observed. Scale bar: 10 μm. (**D**) The apical region of the cells presented a reduced number of blebs, and a low content of secretory granules was observed. Secretions were observed mainly extracellularly. Scale bar: 5 μm; higher magnification, 0.5 μm. (**E**) Extracellular MVB-like EVs were detected outside of the cells, and some were still connected to the bleb cell membrane (blebs). Scale bar: 0.5 μm. (**F** and **G**) In ependymal cells, some cilia showed axonemal bleb-like structures (inset and arrows). Scale bar: 0.5 μm; higher magnification, 0.2 μm. *N* = 5. BB, basal body; CSF, cerebrospinal fluid; EV, extracellular vesicle; MVB, multivesicular body; SCO, subcommissural organ; TEM, transmission electron microscopy.
(TIF)

S4 Fig β-catenin expression and distribution are different in rat and mouse SCO cells. (**A**) Immunohistochemical staining of SCO-Spondin and β-catenin in frontal brain sections from normoglycemic rats. In SCO cells, β-catenin was mainly located in the cellular membrane in the basal area and apical region. Scale bar: 30 μm. (**B**) TEM analysis of the brains of normoglycemic rats. Zonula adherens junctions were mainly detected in the basal and apical regions of the cells (arrows). Scale bar: 1 μm; higher magnification, 0.6 μm. (**C**) Immunohistochemical staining of SCO-Spondin and β-catenin in frontal brain sections from hyperglycemic rats (CSF glucose concentration of 10 mM). In SCO cells, β-catenin was mainly located in the cellular membrane in the basal area and apical region. In the ependyma, β-catenin was mainly detected in the lateral membranes of the cells. Additionally, SCO-Spondin was detected in the apex of ependymal cells cilia. Scale bar: 30 μm. (**D**) Immunohistochemical staining of SCO-Spondin and β-catenin in frontal brain sections from hyperglycemic mice. In SCO cells, β-catenin was located at all the cellular borders, including the basal, lateral, and apical membranes. Scale bar: 30 μm. (**E** and **F**) TEM analysis of the brains of hyperglycemic animals. Zonula adherens junctions were detected in the cell membrane throughout the cell and in the basal, medial, and apical membranes (arrows). Scale bar: E, 5 μm; medial zones, 2 μm; higher magnification, 0.5 μm. *N* = 3. ER, endoplasmic reticulum; SCO, subcommissural organ; TEM, transmission electron microscopy.
(TIF)

S5 Fig SCO cells from old animals do not release SCO-Spondin under hyperglycemic conditions. (**A**) Immunohistochemical staining of GLUT2, SCO-Spondin, acetylated α-tubulin, and vimentin in frontal brain sections from old hyperglycemic rats. Scale bar: 30 μm. (**B**) Immunohistochemical staining of vimentin of frontal brain sections containing ependymal cells from old hyperglycemic rats. Scale bar: 20 μm. (**C**) TEM analysis of SCO cells from hyperglycemic animals. ER and apSGs were abundant. Aggregated SCO-spondin that formed pre-RF was detected extracellularly (inset). No MVBs were observed. Scale bar: 10 μm; ER and apSGs, 2 μm. (**D**) TEM analysis of hyperglycemic SCO cells at high magnification. apSGs were observed in the apical region of cells with a lower content of blebs. Scale bar: 0.5 μm. (**E**) Ependymal cells did not show structural alterations; however, electrolucent areas were detected close to BVs, suggesting perivascular edema. No aggregate secretory material was observed to interact with the apex of ciliated cells, and MVBs were not observed. Scale bar: 5 μm; higher magnification, 2 μm. *N* = 3. apSG, apical secretory granule; BV, blood vessel; CSF, cerebrospinal fluid; ER, endoplasmic reticulum; MVB, multivesicular body; RF, Reissner’s fibers; SCO, subcommissural organ; TEM, transmission electron microscopy.
(TIF)

S6 Fig The ciliary orientation angle of ependymal cells is different in hyperglycemic or normoglycemic conditions. (**A**) Scanning electron microscopy of the dorsal and ventral region (tanycytes) of the third ventricle (colored area) in normoglycemia, observed with low magnification. Zones similar to the areas represented by discolored squares are observed with higher magnification in the lateral images. (**B**) Scanning electron microscopy of the dorsal and ventral region (tanycytes) of the third ventricle (colored area) in hyperglycemia (10 mM glucose in CSF), observed with low magnification. Zones similar to the areas represented by discolored squares are observed with higher magnification in the lateral images. CSF, cerebrospinal fluid.
(TIF)

S7 Fig Wnt5a is detected in dorsal ependymal cells and colocalized with SCO-spondin. (**A**) Immunoperoxidase analysis using an anti-Wnt5a antibody in dorsal ependymal cells in the third ventricle, tanycytes in the ventral hypothalamus, ependymal cells in the lateral ventricle, and choroid plexus cells under hyperglycemic conditions. Scale bar: 25 μm. (**B**) Immunofluorescence staining of Wnt5a and SCO-spondin in dorsal ependymal cell sections under hyperglycemic conditions and confocal analysis. The images represent replicate analysis of the data in Fig 7D. Scale bar: 25 μm. d3v, dorsal third ventricle; LV, lateral ventricle; SCO, subcommissural organ.
(TIF)

S8 Fig ROR2 expression and distribution in ependymal cells. (**A-C**) Immunohistochemical staining of SCO-Spondin and ROR2 in frontal brain sections containing SCO cells and ependymal cells from normoglycemic and hyperglycemic animals. Scale bar: 20 μm. ROR2 was not expressed in SCO cells. Under normoglycemic conditions, ROR2 showed mostly focal immunoreactivity and was internalized in ependymal cells (immunofluorescence and DIC images) (A). However, under hyperglycemic conditions, ROR2 immunoreactivity was detected mainly in the apical membrane of ependymal cells and intracellularly (B and C, areas 1–3). Scale bar: 20 μm. CSF, cerebrospinal fluid; DIC, differential interference contrast; d3v, dorsal third ventricle; ROR2, Frizzled 2/receptor tyrosine kinase-like orphan receptor-2; SCO, subcommissural organ.
(TIF)

S9 Fig Testican and glypican are expressed in ependymal cells. (**A)** Immunohistochemical staining of HS6ST1, aggrecan, and syndecan. Only HS6ST1 was detected in ependymal cells under normoglycemic conditions. Scale bar: 20 μm. (**B-D**) Immunohistochemical staining of SCO-spondin and testican in frontal brain sections from normoglycemic and hyperglycemic rats. Testican was not expressed in SCO cells; however, it was expressed in ependymal cells, without no changes being observed between normoglycemic and hyperglycemic conditions. Scale bar: 30 μm. *N = 3*. (**E-G**) Immunohistochemical staining of glypican in frontal brain sections from normoglycemic and hyperglycemic rats. Glypican was expressed in ependymal cells, without no changes being observed between normoglycemic and hyperglycemic conditions. Scale bar: 30 μm. *N = 3*. CSF, cerebrospinal fluid; HS6ST1, heparan sulfate-6-sulfotransferase 1; SCO, subcommissural organ.
(TIF)

S10 Fig Retrospective analysis of gene expression in EPN cells. (**A**) UMAP of scRNA-seq data from 26 pediatric patients with EPN. Tumor populations are shown in color. There were 7 posterior fossa group A subgroups (PFA-sc1 to PFA-sc7), 5 C11ORF95-RELA subgroups (RELA-sc1 to RELA-sc5), 1 posterior fossa group B group (PFB), and 1 YAP-MAMLD1 group (YAP). The 5 PFA subgroups corresponded to CECs, TECs, MECs, and undifferentiated EPN cells −1 and −2 (UEC-1, UEC-2). Cells in mitosis (mitotic). Gene expression is shown in a range of brown colors and corresponds to the values obtained by ALRA. The database was generated by Gillen and colleagues [41], and the full EPN scRNA-seq dataset is available at the Pediatric Neuro-Oncology Cell Atlas (pneuroonccellatlas.org). (**B**-**P**) Analysis of CECs expressing Wnt5a, Frizzled-3, 5, and 6, ROR2, testican-2, and glypican-1. GLUT1, MCT1, MCT2, and Cx43 were also detected. In addition to Spondin 2 and R-spondin 4, SCO-spondin was detected in a few CECs. ALRA, adaptative thresholder low-rank approximation; CEC, ciliated ependymal cell; EPN, ependymoma; MEC, mesenchymal EPN cell; TEC, transportive EPN cell; UMAP, uniform manifold approximation and projection.
(TIF)

S1 Data Original data for the different graphs. Each tab includes data for individual panels of Figs 1F, 1L, 1W, 1X, 1Y, 1Z, 2K, 2L, 3H, 4L, 5C, 5E, 5I, 5K, 6C, 6D, 6E, 6H, 7F, 7G, 8C, 8F, 8I, 9D, 9E, and 9F.
(XLSX)

S1 Raw Images Original blots for Fig 10F. Dot blots for human R-Spondin 4, SCO-Spondin, and BSA by using anti-SCO-Spondin antibody (Top). Membranes were stained with India ink 0,1% (bottom). Red boxes represent the cropped membranes in Fig 10F.
(TIF)

## Abbreviations

2-NBDG: D-glucose analog 2-[N-(7-nitrobenz-2-oxa-1,3-diazol-4-yl) amino]-2-deoxy-D-glucose
5-HT: 5-hydroxytryptamine
aCSF: artificial CSF
BSA: bovine serum albumin
CA: cerebral aqueduct
CEC: ciliated ependymal cell
CR: collicular recess
CSF: cerebrospinal fluid
Cx43: connexin-43
DIC: differential interference contrast
EPN: ependymoma
ER: endoplasmic reticulum
EV: extracellular vesicle
GFAP: glial fibrillary acidic protein
GLUT: glucose transporter
HS6ST1: heparan sulfate-6-sulfotransferase 1
HSPG: heparan sulfate proteoglycan
IP: intraperitoneal(ly)
MCH: melanin-concentrating hormone
MCT: monocarboxylate transporter
ME: median eminence
MFI: mean fluorescence intensity
MRI: magnetic resonance imaging
MVB: multivesicular body
PAS: Periodic acid–Schiff
RF: Reissner’s fibers
ROI: region of interest
ROR2: Frizzled 2/receptor tyrosine kinase-like orphan receptor-2
RT: room temperature
SCO: subcommissural organ
SV: secretory vesicle
TEM: transmission electron microscopy
TSR: thrombospondin type 1 repeat
VGCC: voltage-gated calcium channel
Wnt5a: wingless-type MMTV integration site family member 5A
