# Supplementary figures and images for: Hyperglycemia increases SCO-spondin and Wnt5a secretion into the cerebrospinal fluid to regulate ependymal cell beating and glucose sensing
Source: PLoS Biol. 2023 Sep 21;21(9):e3002308. doi: 10.1371/journal.pbio.3002308 (PMC10513282; doi:10.1371/journal.pbio.3002308)

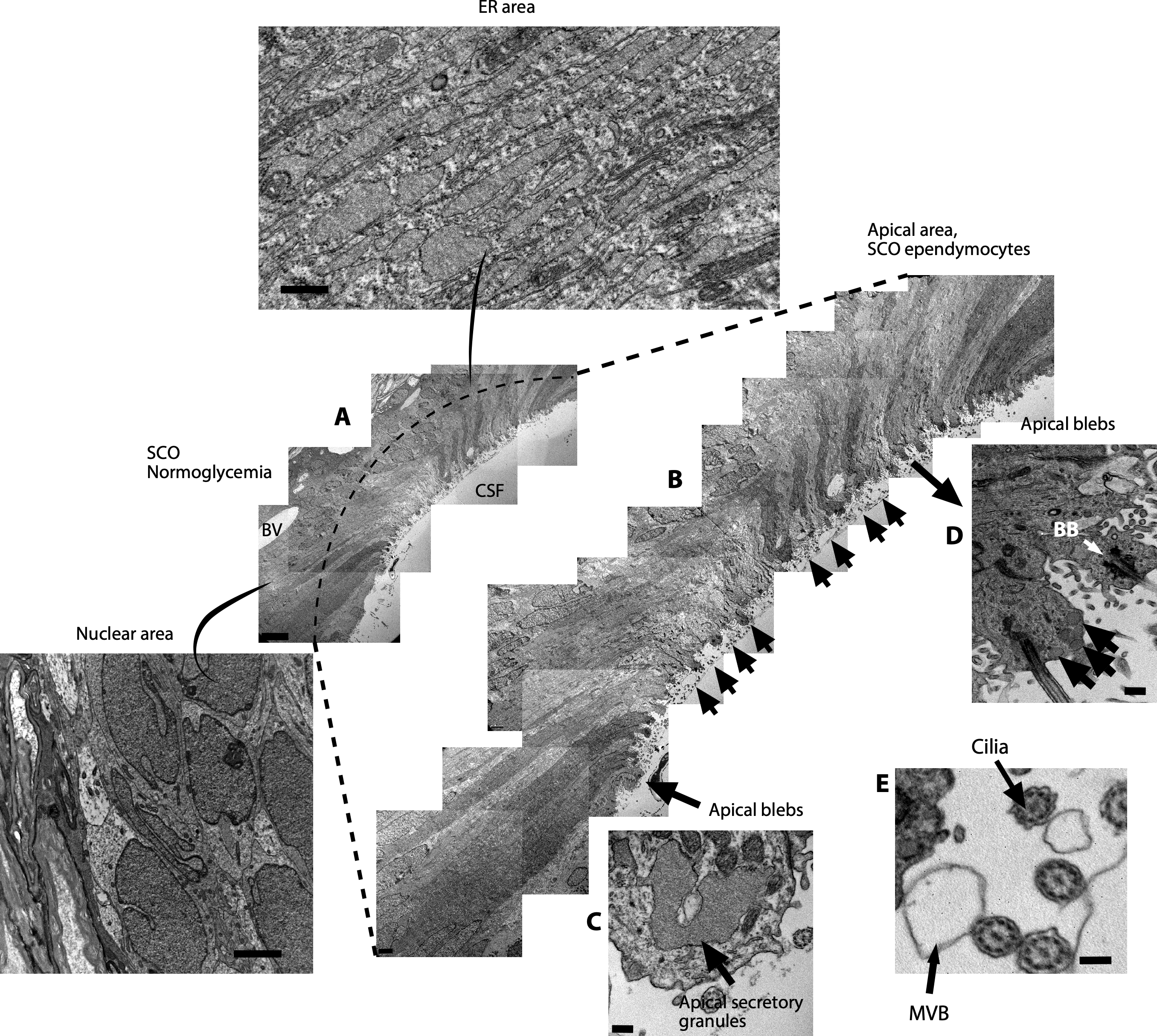

Supplement: S1 Fig — (A, B) Tile-scanning TEM. Under normoglycemic conditions, SCO cells did not exhibit structural changes, were normally polarized, and had dilated ER cisternae (A, ER area), and apical areas with blebs containing secretory granules, microvilli and cilia (B, C, D, black arrows). MVB, most of them containing few vesicles (E), were also detected. Scale bar: A, 10 μm; B, ER and nuclear areas, 2 μm; C to E, 0.2 μm. N = 3. BB, basal body; BV, blood vessel; ER, endoplasmic reticulum; MVB, multivesicular body; SCO, subcommissural organ; TEM, transmission electron microscopy. (TIF) [file pbio.3002308.s001.tif]

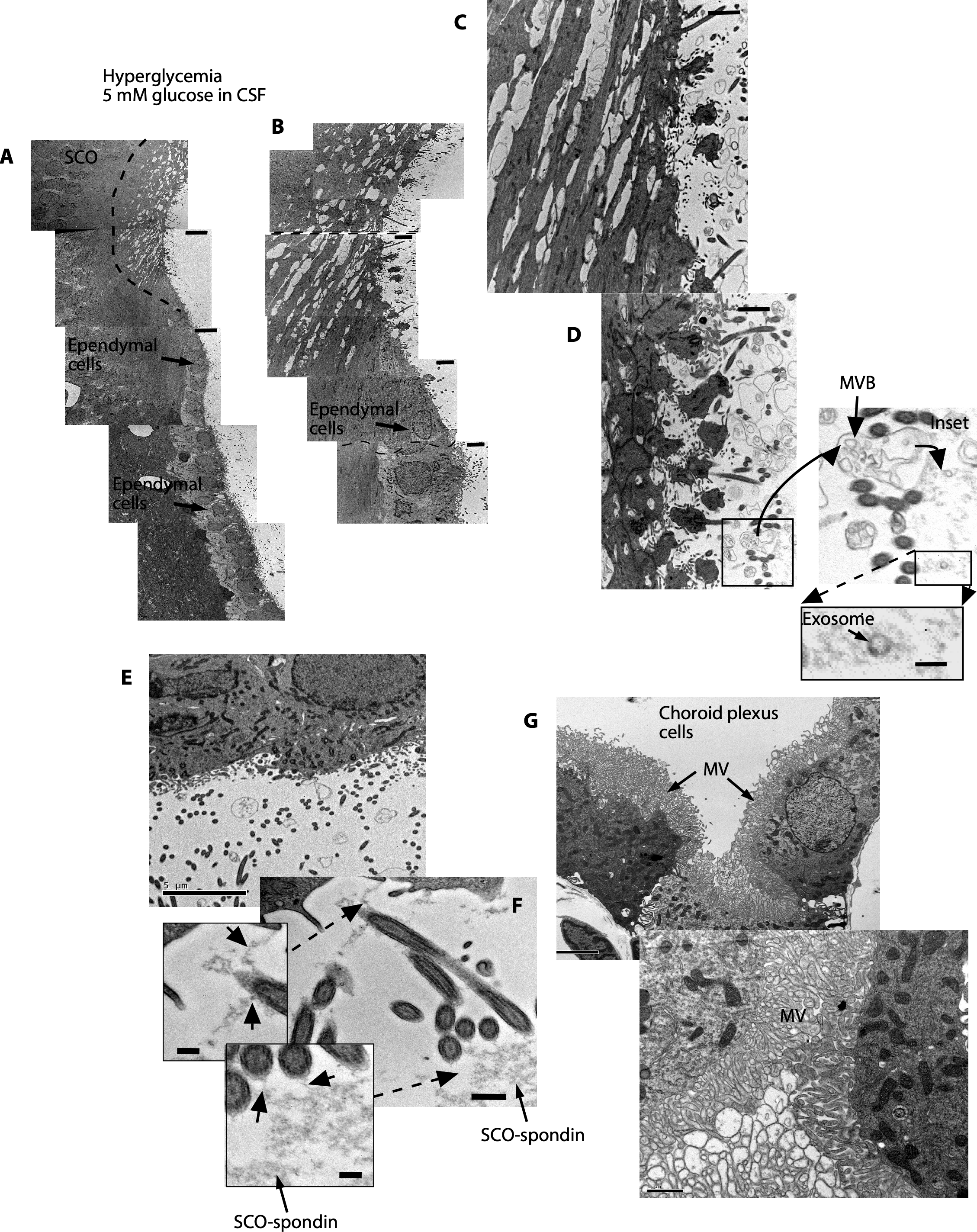

Supplement: S2 Fig — (A-C) TEM analysis when the CSF glucose concentration was 5 mM. SCO cells with constricted morphology and intercellular spaces in areas close to the ventricle were observed. Scale bar: A, 10 μm; B, 2 μm; C, 4 μm. (D) Apical region of SCO cells. We detected blebs, cilia, and microvilli and extracellularly disaggregated SCO-spondin. MVBs-like EV were secreted (D, insets). Exosome-like vesicles intermixed with secreted SCO-Spondin and cilia were also observed (arrows and insets). Scale bar: D, 1 μm; higher magnification, 0.1 μm. (E) Dorsal ependymal cells. Scale bar: 5 μm. (F) Ependymal cell cilia. Floccular material associated with cilia membranes (arrow and insets). Scale bar: 0.5 μm; higher magnification, 0.2 μm. (G) Choroidal plexus cells showed a normal structure without secretory material outside the cells. Scale bar: 3 μm; higher magnification, 1 μm. N = 4. CSF, cerebrospinal fluid; EV, extracellular vesicle; MV, microvilli; MVB, multivesicular body; SCO, subcommissural organ; TEM, transmission electron microscopy. (TIF) [file pbio.3002308.s002.tif]

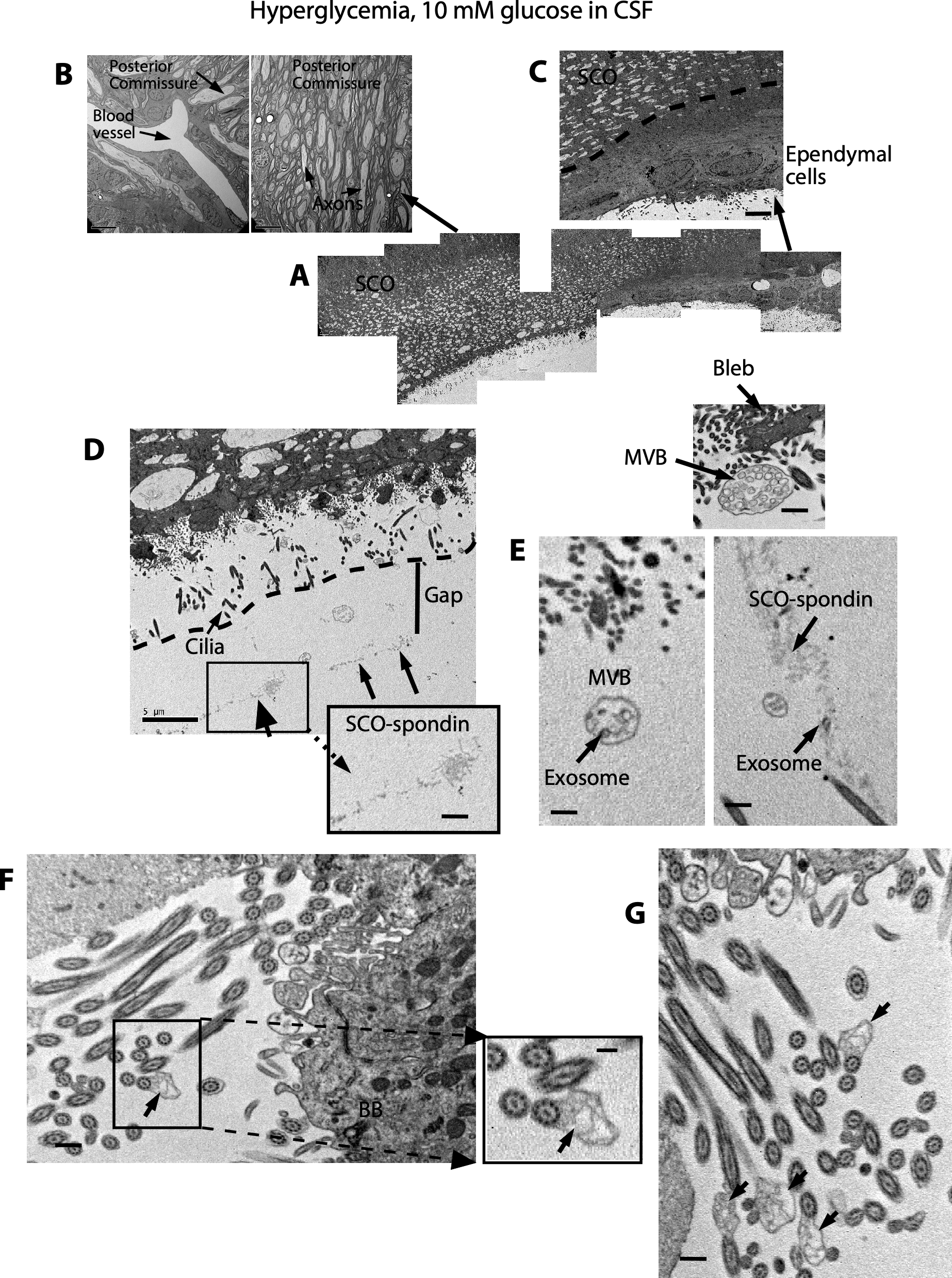

Supplement: S3 Fig — (A) Tile-scanning TEM. SCO cells were contraction, giving the impression of a “pricked” tissue. Scale bar: A, 5 μm. (B and C) TEM revealed no apparent structural changes in the posterior commissure (axons or blood vessels) or ependymal cells. No subependymal edema was observed. Scale bar: 10 μm. (D) The apical region of the cells presented a reduced number of blebs, and a low content of secretory granules was observed. Secretions were observed mainly extracellularly. Scale bar: 5 μm; higher magnification, 0.5 μm. (E) Extracellular MVB-like EVs were detected outside of the cells, and some were still connected to the bleb cell membrane (blebs). Scale bar: 0.5 μm. (F and G) In ependymal cells, some cilia showed axonemal bleb-like structures (inset and arrows). Scale bar: 0.5 μm; higher magnification, 0.2 μm. N = 5. BB, basal body; CSF, cerebrospinal fluid; EV, extracellular vesicle; MVB, multivesicular body; SCO, subcommissural organ; TEM, transmission electron microscopy. (TIF) [file pbio.3002308.s003.tif]

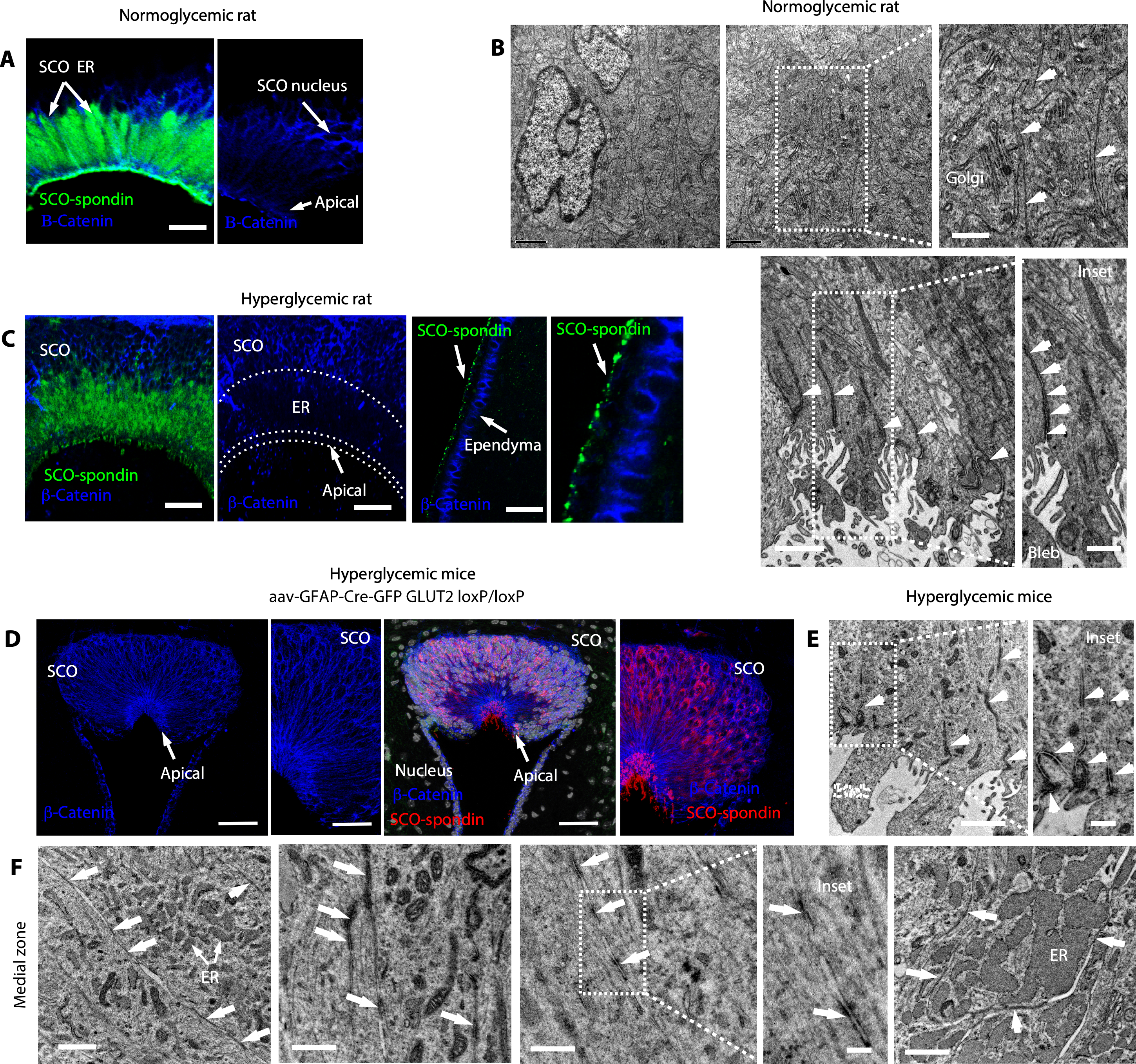

Supplement: S4 Fig — (A) Immunohistochemical staining of SCO-Spondin and β-catenin in frontal brain sections from normoglycemic rats. In SCO cells, β-catenin was mainly located in the cellular membrane in the basal area and apical region. Scale bar: 30 μm. (B) TEM analysis of the brains of normoglycemic rats. Zonula adherens junctions were mainly detected in the basal and apical regions of the cells (arrows). Scale bar: 1 μm; higher magnification, 0.6 μm. (C) Immunohistochemical staining of SCO-Spondin and β-catenin in frontal brain sections from hyperglycemic rats (CSF glucose concentration of 10 mM). In SCO cells, β-catenin was mainly located in the cellular membrane in the basal area and apical region. In the ependyma, β-catenin was mainly detected in the lateral membranes of the cells. Additionally, SCO-Spondin was detected in the apex of ependymal cells cilia. Scale bar: 30 μm. (D) Immunohistochemical staining of SCO-Spondin and β-catenin in frontal brain sections from hyperglycemic mice. In SCO cells, β-catenin was located at all the cellular borders, including the basal, lateral, and apical membranes. Scale bar: 30 μm. (E and F) TEM analysis of the brains of hyperglycemic animals. Zonula adherens junctions were detected in the cell membrane throughout the cell and in the basal, medial, and apical membranes (arrows). Scale bar: E, 5 μm; medial zones, 2 μm; higher magnification, 0.5 μm. N = 3. ER, endoplasmic reticulum; SCO, subcommissural organ; TEM, transmission electron microscopy. (TIF) [file pbio.3002308.s004.tif]

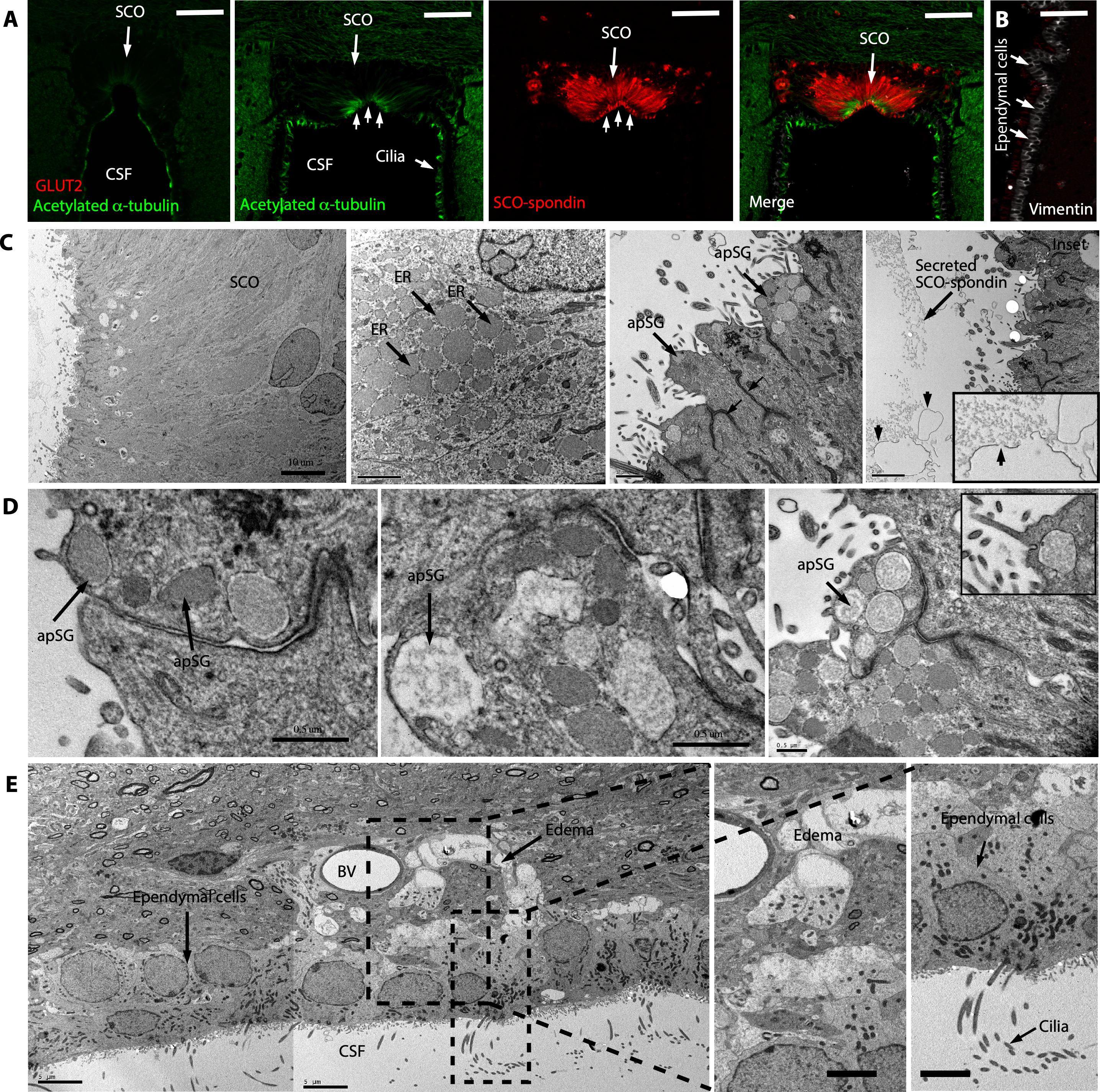

Supplement: S5 Fig — (A) Immunohistochemical staining of GLUT2, SCO-Spondin, acetylated α-tubulin, and vimentin in frontal brain sections from old hyperglycemic rats. Scale bar: 30 μm. (B) Immunohistochemical staining of vimentin of frontal brain sections containing ependymal cells from old hyperglycemic rats. Scale bar: 20 μm. (C) TEM analysis of SCO cells from hyperglycemic animals. ER and apSGs were abundant. Aggregated SCO-spondin that formed pre-RF was detected extracellularly (inset). No MVBs were observed. Scale bar: 10 μm; ER and apSGs, 2 μm. (D) TEM analysis of hyperglycemic SCO cells at high magnification. apSGs were observed in the apical region of cells with a lower content of blebs. Scale bar: 0.5 μm. (E) Ependymal cells did not show structural alterations; however, electrolucent areas were detected close to BVs, suggesting perivascular edema. No aggregate secretory material was observed to interact with the apex of ciliated cells, and MVBs were not observed. Scale bar: 5 μm; higher magnification, 2 μm. N = 3. apSG, apical secretory granule; BV, blood vessel; CSF, cerebrospinal fluid; ER, endoplasmic reticulum; MVB, multivesicular body; RF, Reissner’s fibers; SCO, subcommissural organ; TEM, transmission electron microscopy. (TIF) [file pbio.3002308.s005.tif]

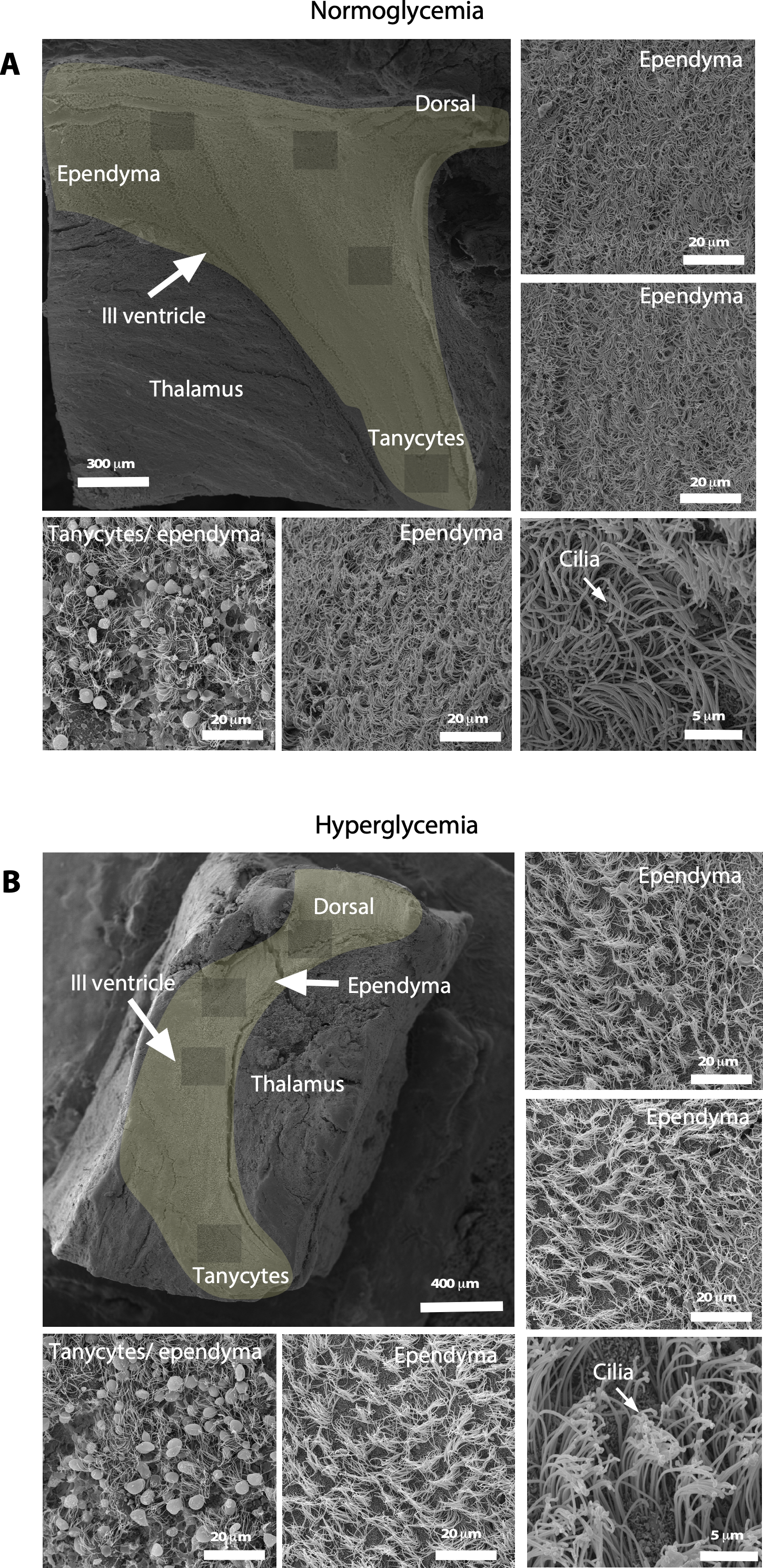

Supplement: S6 Fig — (A) Scanning electron microscopy of the dorsal and ventral region (tanycytes) of the third ventricle (colored area) in normoglycemia, observed with low magnification. Zones similar to the areas represented by discolored squares are observed with higher magnification in the lateral images. (B) Scanning electron microscopy of the dorsal and ventral region (tanycytes) of the third ventricle (colored area) in hyperglycemia (10 mM glucose in CSF), observed with low magnification. Zones similar to the areas represented by discolored squares are observed with higher magnification in the lateral images. CSF, cerebrospinal fluid. (TIF) [file pbio.3002308.s006.tif]

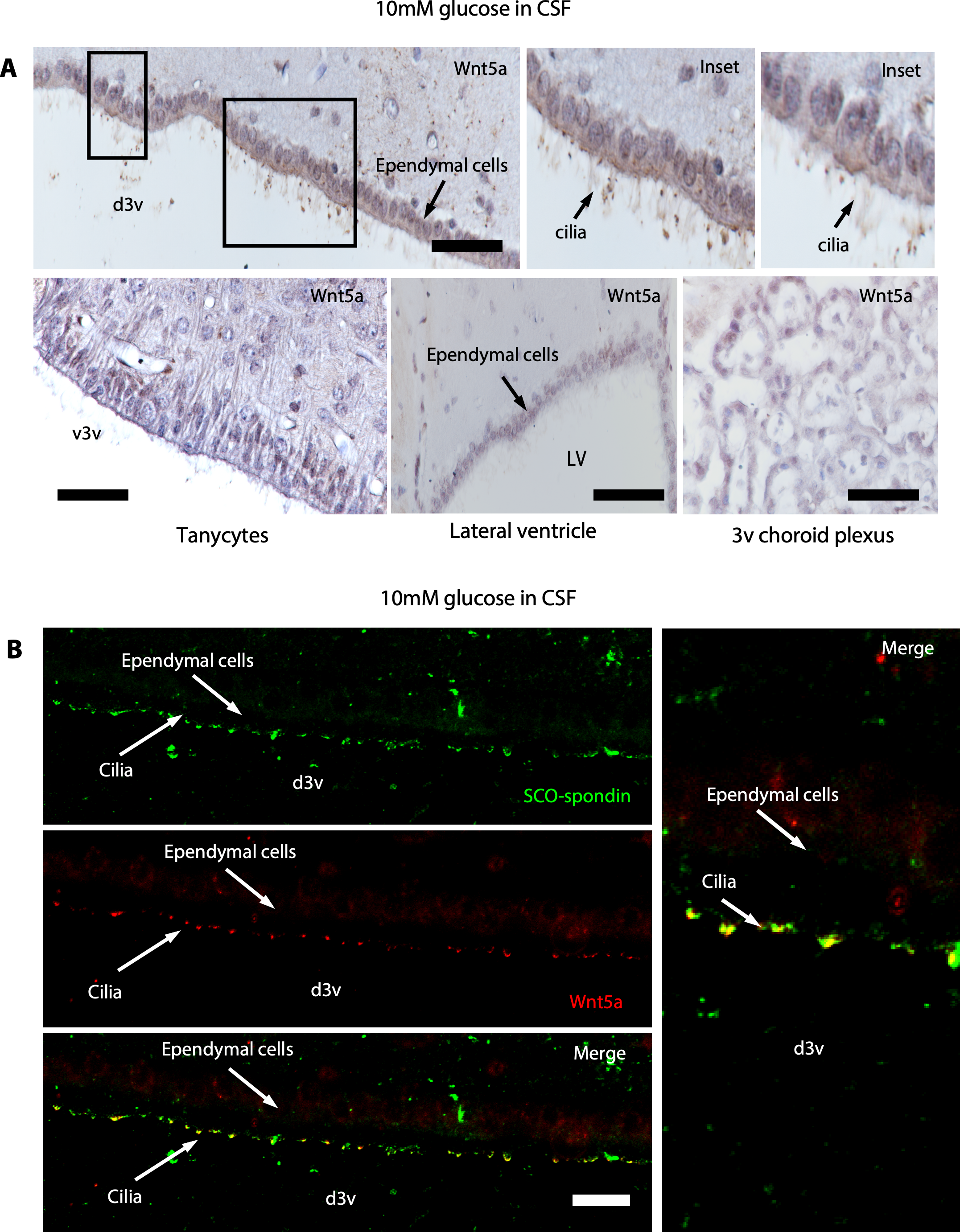

Supplement: S7 Fig — (A) Immunoperoxidase analysis using an anti-Wnt5a antibody in dorsal ependymal cells in the third ventricle, tanycytes in the ventral hypothalamus, ependymal cells in the lateral ventricle, and choroid plexus cells under hyperglycemic conditions. Scale bar: 25 μm. (B) Immunofluorescence staining of Wnt5a and SCO-spondin in dorsal ependymal cell sections under hyperglycemic conditions and confocal analysis. The images represent replicate analysis of the data in Fig 7D. Scale bar: 25 μm. d3v, dorsal third ventricle; LV, lateral ventricle; SCO, subcommissural organ. (TIF) [file pbio.3002308.s007.tif]

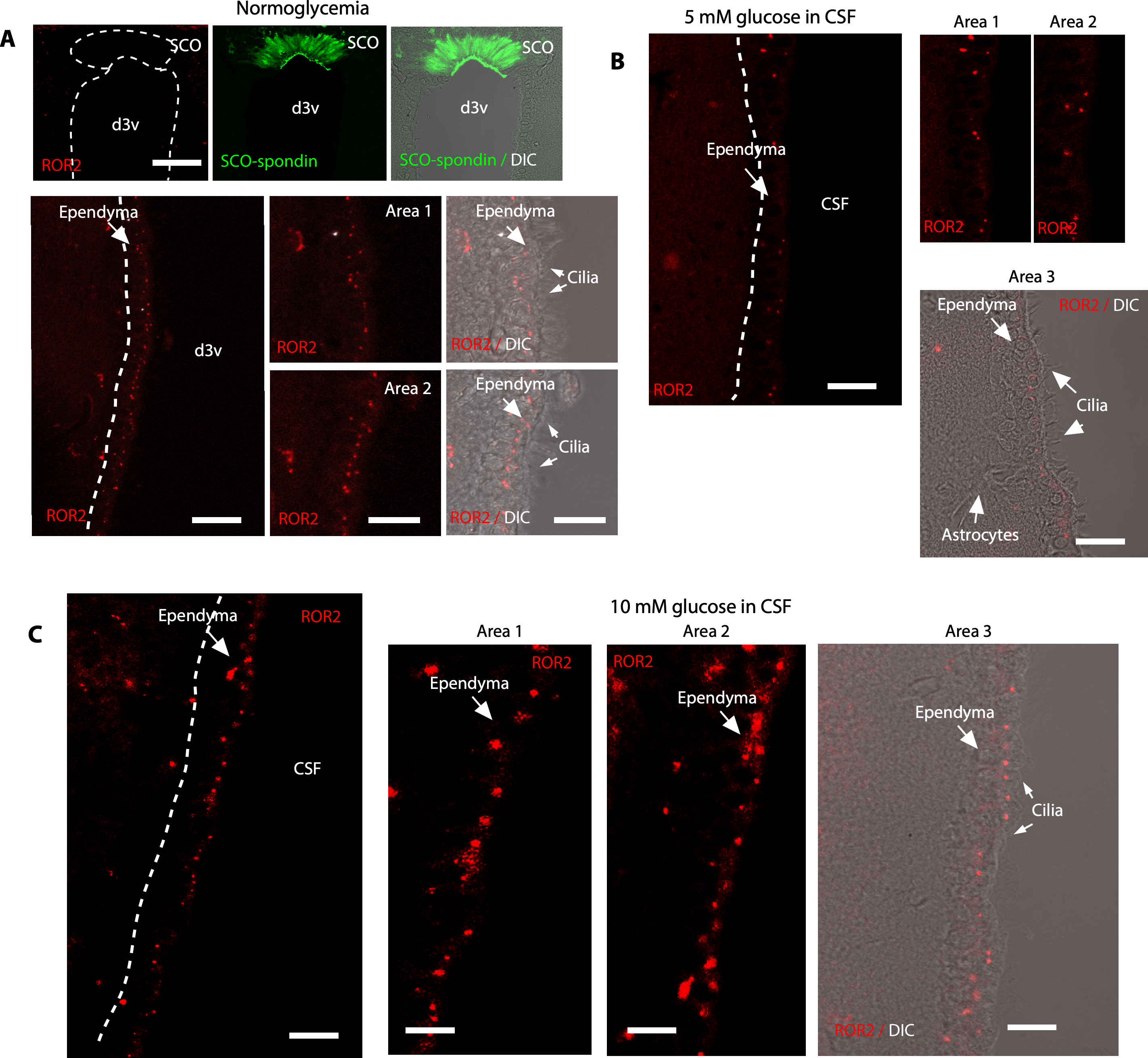

Supplement: S8 Fig — (A-C) Immunohistochemical staining of SCO-Spondin and ROR2 in frontal brain sections containing SCO cells and ependymal cells from normoglycemic and hyperglycemic animals. Scale bar: 20 μm. ROR2 was not expressed in SCO cells. Under normoglycemic conditions, ROR2 showed mostly focal immunoreactivity and was internalized in ependymal cells (immunofluorescence and DIC images) (A). However, under hyperglycemic conditions, ROR2 immunoreactivity was detected mainly in the apical membrane of ependymal cells and intracellularly (B and C, areas 1–3). Scale bar: 20 μm. CSF, cerebrospinal fluid; DIC, differential interference contrast; d3v, dorsal third ventricle; ROR2, Frizzled 2/receptor tyrosine kinase-like orphan receptor-2; SCO, subcommissural organ. (TIF) [file pbio.3002308.s008.tif]

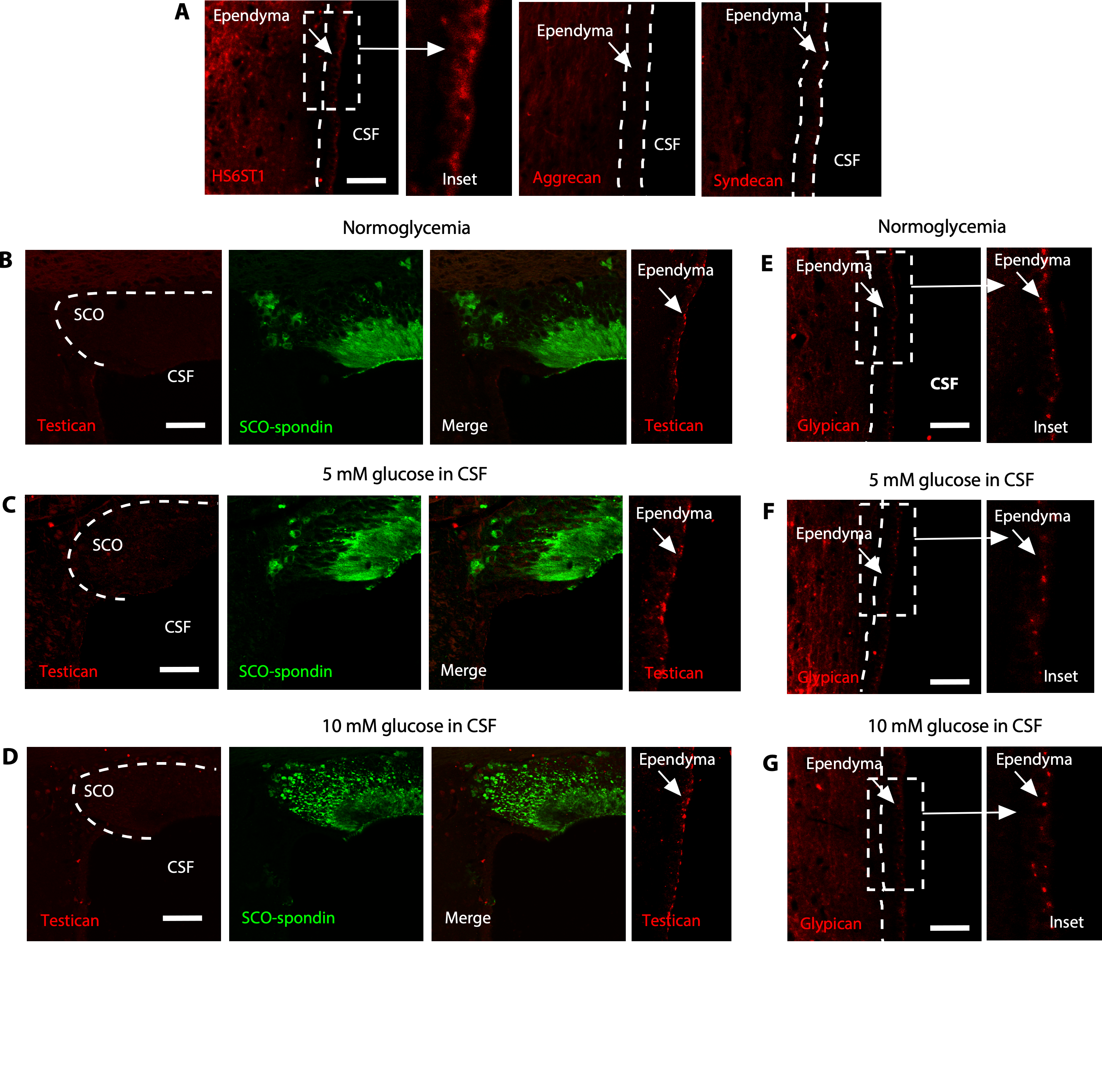

Supplement: S9 Fig — (A) Immunohistochemical staining of HS6ST1, aggrecan, and syndecan. Only HS6ST1 was detected in ependymal cells under normoglycemic conditions. Scale bar: 20 μm. (B-D) Immunohistochemical staining of SCO-spondin and testican in frontal brain sections from normoglycemic and hyperglycemic rats. Testican was not expressed in SCO cells; however, it was expressed in ependymal cells, without no changes being observed between normoglycemic and hyperglycemic conditions. Scale bar: 30 μm. N = 3. (E-G) Immunohistochemical staining of glypican in frontal brain sections from normoglycemic and hyperglycemic rats. Glypican was expressed in ependymal cells, without no changes being observed between normoglycemic and hyperglycemic conditions. Scale bar: 30 μm. N = 3. CSF, cerebrospinal fluid; HS6ST1, heparan sulfate-6-sulfotransferase 1; SCO, subcommissural organ. (TIF) [file pbio.3002308.s009.tif]

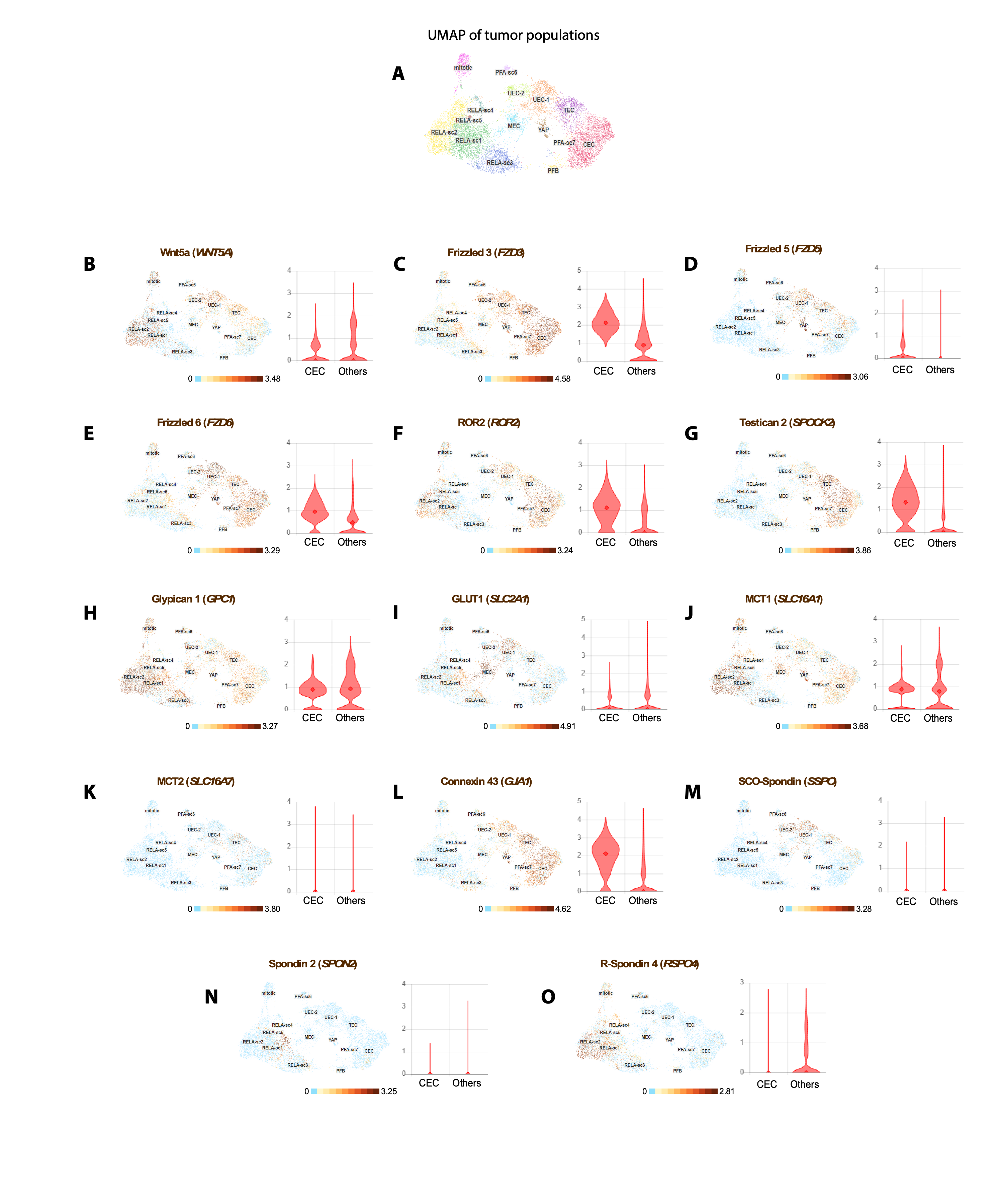

Supplement: S10 Fig — (A) UMAP of scRNA-seq data from 26 pediatric patients with EPN. Tumor populations are shown in color. There were 7 posterior fossa group A subgroups (PFA-sc1 to PFA-sc7), 5 C11ORF95-RELA subgroups (RELA-sc1 to RELA-sc5), 1 posterior fossa group B group (PFB), and 1 YAP-MAMLD1 group (YAP). The 5 PFA subgroups corresponded to CECs, TECs, MECs, and undifferentiated EPN cells −1 and −2 (UEC-1, UEC-2). Cells in mitosis (mitotic). Gene expression is shown in a range of brown colors and corresponds to the values obtained by ALRA. The database was generated by Gillen and colleagues [41], and the full EPN scRNA-seq dataset is available at the Pediatric Neuro-Oncology Cell Atlas (pneuroonccellatlas.org). (B-P) Analysis of CECs expressing Wnt5a, Frizzled-3, 5, and 6, ROR2, testican-2, and glypican-1. GLUT1, MCT1, MCT2, and Cx43 were also detected. In addition to Spondin 2 and R-spondin 4, SCO-spondin was detected in a few CECs. ALRA, adaptative thresholder low-rank approximation; CEC, ciliated ependymal cell; EPN, ependymoma; MEC, mesenchymal EPN cell; TEC, transportive EPN cell; UMAP, uniform manifold approximation and projection. (TIF) [file pbio.3002308.s010.tif]

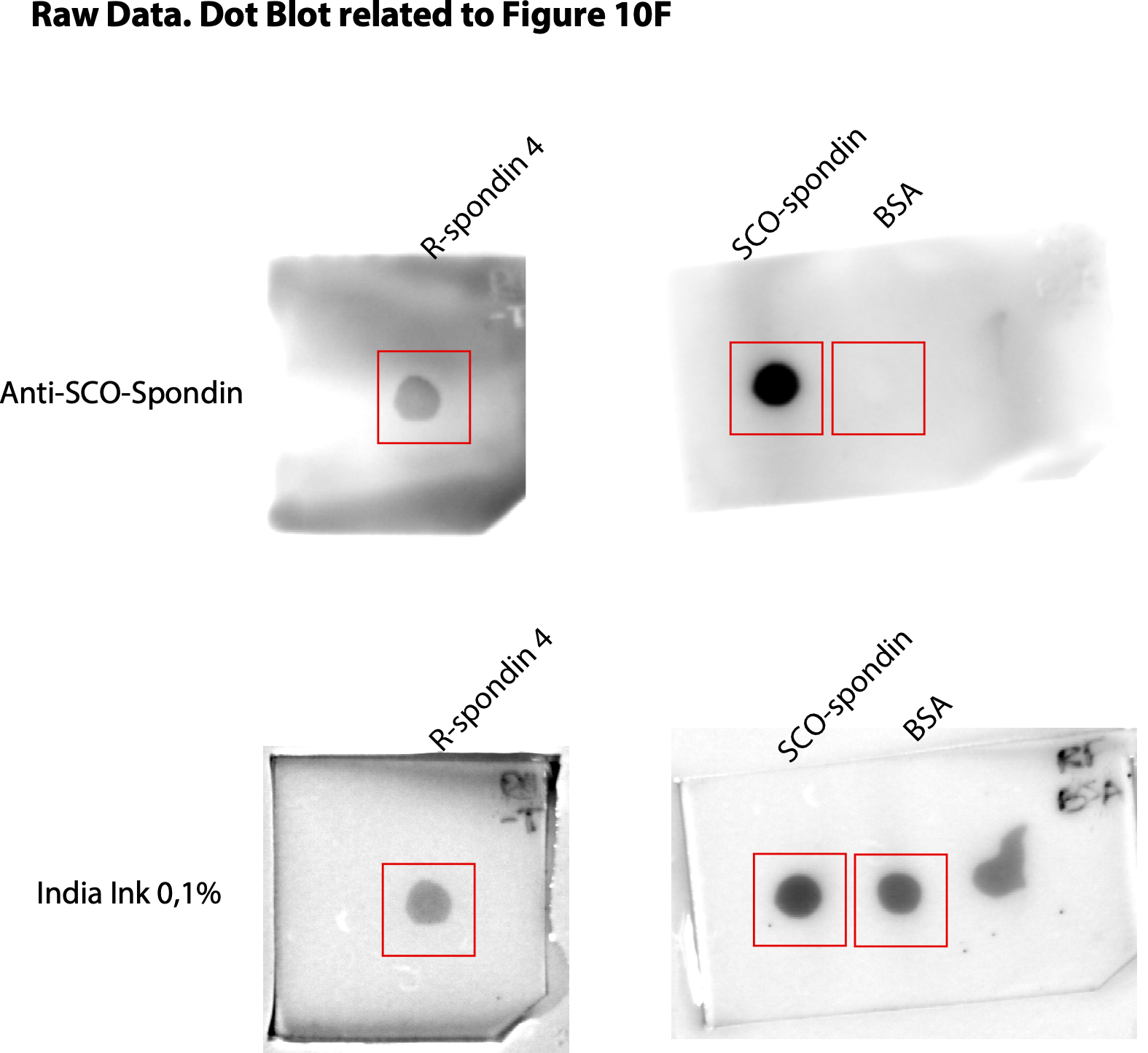

Supplement: S1 Raw Images — Dot blots for human R-Spondin 4, SCO-Spondin, and BSA by using anti-SCO-Spondin antibody (Top). Membranes were stained with India ink 0,1% (bottom). Red boxes represent the cropped membranes in Fig 10F. (TIF) [file pbio.3002308.s012.tif]
